# Optimal vaccination policy to prevent endemicity: a stochastic model

**DOI:** 10.1007/s00285-024-02171-z

**Published:** 2024-12-19

**Authors:** Félix Foutel-Rodier, Arthur Charpentier, Hélène Guérin

**Affiliations:** 1 Department of Statistics, University of Oxford, Oxford, UK; 2 Département de Mathématiques, Université du Québec à Montréal, Montréal, Canada

**Keywords:** Age-structured model, Endemicity, Waning immunity, Heterogeneous vaccination, Varying infectiousness and susceptibility, Mitigation, Non-Markovian model, Recurrent vaccination, Primary: 92D30 Secondary: 60F17, 35Q92

## Abstract

We examine here the effects of recurrent vaccination and waning immunity on the establishment of an endemic equilibrium in a population. An individual-based model that incorporates memory effects for transmission rate during infection and subsequent immunity is introduced, considering stochasticity at the individual level. By letting the population size going to infinity, we derive a set of equations describing the large scale behavior of the epidemic. The analysis of the model’s equilibria reveals a criterion for the existence of an endemic equilibrium, which depends on the rate of immunity loss and the distribution of time between booster doses. The outcome of a vaccination policy in this context is influenced by the efficiency of the vaccine in blocking transmissions and the distribution pattern of booster doses within the population. Strategies with evenly spaced booster shots at the individual level prove to be more effective in preventing disease spread compared to irregularly spaced boosters, as longer intervals without vaccination increase susceptibility and facilitate more efficient disease transmission. We provide an expression for the critical fraction of the population required to adhere to the vaccination policy in order to eradicate the disease, that resembles a well-known threshold for preventing an outbreak with an imperfect vaccine. We also investigate the consequences of unequal vaccine access in a population and prove that, under reasonable assumptions, fair vaccine allocation is the optimal strategy to prevent endemicity.

## Introduction

In epidemiology, a disease is called endemic if it persists in a population over a long period of time. Many diseases are endemic in some parts of the world, including for instance malaria and tuberculosis (Hay et al. 2009; Oliwa et al. 2015), and several studies have proposed that endemicity is a likely outcome for the recent COVID-19 epidemic (Antia and Halloran 2021; Lavine et al. 2021), as is currently the case for other human coronavirus-induced diseases (Shuo et al. 2016). The persistence of a disease in a population can incur a large cost for society and endemic diseases are responsible for a large share of the deaths from communicable diseases every year. Understanding the mechanisms underlying the establishment of such an endemic state and how to control it is therefore of great public health importance. Prophylactic vaccination, when available, is a common and efficient way to mitigate the spread of diseases (Plotkin 2005; Rashid et al. 2012). If the vaccine blocks part of the transmissions, a high enough vaccine coverage can prevent self-sustained transmissions in the population, leading to a so-called herd immunity (Anderson and May 1985; Fine et al. 2011; Randolph and Barreiro 2020). This phenomenon has been the subject of a large body of work in the mathematical modeling literature, aimed at informing policy-makers on the effectiveness of a vaccination campaign and at developing a theoretical understanding of the epidemiological consequences of vaccination. An important achievement of these studies is the derivation of an expression for the critical vaccine coverage required to eradicate a disease, under various scenarios of increasing complexity (Anderson and May 1982; Farrington 2003; Magpantay 2017; Delmas et al. 2022). However, the bulk of this work pertains to vaccines providing life-long (or slowly waning) immunity and administrated at birth or at a single point in time. Although these assumptions might represent adequately many situations (including for instance childhood diseases), infection by some pathogens and vaccines are known to provide no or temporary immunity (Vynnycky and Fine 1997; RTS 2005; Stein et al. 2023). An important motivating example for our work is the recent COVID-19 epidemic, for which reinfections after either primary infection or vaccination have been reported (Stein et al. 2023), and for which direct measurements of several components of adaptive immunity suggest that part of it is waning (Shrotri et al. 2022; Lin et al. 2022). The understanding of the impact of vaccination under such short-lived immunity remains limited and motivates further theoretical developments.

In this work, we consider a pathogen for which a vaccine that blocks transmissions is available but with an immunity that wanes with time, both for individuals infected and vaccinated. Although our main motivation is COVID-19, we consider a generic disease with these two features. As the immunity conferred by the vaccine is temporary, the effect of a single vaccination rapidly fades and herd immunity can only be achieved (and thus an endemic state prevented) if individuals are vaccinated recurrently (Randolph and Barreiro 2020). However, even recurrent vaccination might fail to provide herd immunity. Under recurrent vaccination, the level of immunity in the population is shaped by two antagonistic forces: boosting due to vaccine injections and re-exposure to the pathogen, and waning due to decay in circulating antibody levels and/or memory cells. If vaccination is too scarce or immunity decays too rapidly, the vaccine-induced immunity might not block enough transmissions to prevent the disease from spreading in the population and reaching endemicity. What drives the outcome of a vaccination policy is therefore a complex interplay between the transmissibility of the disease, the waning of the immunity (which sets up the time scale after which reinfection can occur) and the frequency of immune boosting by vaccines. We investigate this effect by constructing an epidemic model that incorporates both waning immunity and recurrent vaccination, and by analysing how these two components interact to determine the long-term establishment of the disease.

In standard SIR-type models, waning immunity can be modeled by letting the infected individuals go back to a susceptible state, either directly after the infection as in the SIS model, or after a temporary immune period as in the SIRS model (Brauer et al. 2019). Extensions of these models where the duration of the immune period is fixed or has a general distribution have also been proposed, for instance in Hethcote et al. (1981); Cooke and Van Den Driessche (1996); Taylor and Carr (2009); Bhattacharya and Adler (2012). In such models, immunity is lost instantaneously as individuals go from being fully protected (in the *R* state) to being fully susceptible to the disease (in the *S* state). Some studies consider a more gradual loss of immunity by adding one or several intermediate compartments with partial immunity, often denoted by *W* (for waning) (Lavine et al. 2011; Carlsson et al. 2020). In our work, we will model the decay of immunity by tracking for each individual a *susceptibility* giving the probability of being reinfected upon exposure to the pathogen. Waning immunity is modeled by having the susceptibility increase with time following an infection or vaccination, with no further assumption. This approach can account for the situations described above, where each individual is in one of finitely many immune states (*R*, *S*, *W*), but also for a continuous loss of immunity. The idea of modeling a susceptibility dates back to the endemic models of Kermack and McKendrick (Kermack and McKendrick 1932, 1933), see also Inaba (2001); Breda et al. (2012) for modern formulations, and is also reminiscent of existing works describing immunity as a continuous variable (White and Medley 1998; Diekmann et al. 2018; Barbarossa and Röst 2015; Martcheva and Pilyugin 2006). Modeling immunity through an abstract susceptibility is a phenomenological approach, but mechanistic approaches have also been proposed. These require to model explicitly for each individual some components of the immune system (T-cells, B-cells, antibodies, cytokines) and their interaction with the pathogen, as for instance in Heffernan and Keeling (2008, 2009); Ashish Goyal et al. (2020); Néant et al. (2021). Such an approach is both more realistic and opens the possibility of being calibrated using clinical data (Lin et al. 2022), but adds a new layer of complexity (the within-host dynamics) which can be cumbersome for theory purpose. We will think of our susceptibility as aggregating the effect of this complicated within-host process.

The effect of immune boosting through recurrent vaccination has also drawn attention from modelers (Julien Arino et al. 2003; Lavine et al. 2011; Carlsson et al. 2020; Leung et al. 2018). Let us note that, in the vaccination strategy that we consider, individuals are vaccinated recurrently during their lifetime, each at different moments. The name “continuous vaccination” has also been proposed for this type of vaccination (Liu et al. 2008; Li and Yang 2011). This is different for instance of the “pulse vaccination strategy”, considered in Agur et al. (1993) in the context of measles, and that has received further attention (Liu et al. 2008; Li and Yang 2011). In this strategy, at several fixed moments a fraction of the population is vaccinated, all individuals in a given vaccination pulse receiving their dose at the same time. Typically, recurrent boosting is modeled by letting individuals get vaccinated at a given rate (that can depend on age or other factors) in which case they are moved to a compartment with a reduced susceptibility. A notable difference with our work is that, in our model, the vaccination rate depends on the time elapsed since the previous vaccination. This reflects the fact that, at the microscopic level, we let the period of time between two consecutive vaccinations have a general (non-exponential) distribution. We have several motivations for relaxing the usual constant rate assumption. First, the resulting dynamics is much richer and complex. It encompasses more realistic situations that cannot be modeled using a constant rate, for instance the enforcement of a minimal duration between two vaccine doses, or the existence of a typical duration between two doses, leading to a peak in the distribution of this duration. Second, summarizing the effect of vaccination and waning immunity by a small number of parameters (the transition rates between the various immune compartments) obscures the role played by the exact shapes of the immunity decay and of the distribution between vaccine doses and leads to quite opaque expressions. For instance, if immunity following vaccination first plateaus and is then lost rapidly around a typical time, we expect a population where boosting occurs right before this time to build a much stronger immunity than if boosting occurred right after this time, although there is only a minimal variation in the overall vaccination rate. This type of effect cannot be studied by assuming that all durations have exponential distributions. From a mathematical point of view, this more general model requires to work with partial differential equations describing the “age structure” of the population rather than with more usual sets of ordinary differential equations. It is always interesting to note that similar age-structured epidemic models were used as early as in the foundational work of Kermack and McKendrick (Kermack and McKendrick 1927), and that the compartmental SIR model was only introduced as a particular case of this more general dynamics.

Our model has one last specificity compared to more classical approaches based on compartments. It is formulated as an individual-based stochastic model from which we derive a set of deterministic equations describing its scaling limit (as the population size goes to infinity). Modeling the population at the microscopic level rather than directly at the continuum gives a better understanding of the hypotheses underlying the model, as well as a more transparent interpretation of the parameters as individual quantities. Moreover, in our model, part of this stochasticity will remain in the limit (through a conditioning term) which could have been easily missed if this convergence step was not carried out. Similar laws of large numbers have been obtained frequently in the probabilistic literature on population models (Thomas 1981; Oelschlager 1990; Fournier and Méléard 2004), in particular in an epidemic context (Clémençon et al. 2008; Barbour and Reinert 2013; Britton et al. 2019; Pang and Pardoux 2023; Foutel-Rodier et al. 2022). Our model draws inspiration from recent works on similar non-Markovian epidemics (Pang and Pardoux 2023; Forien et al. 2021; Foutel-Rodier et al. 2022; Duchamps et al. 2023), and the limiting equations we obtain are connected to classical time-since-infection models in epidemiology (Diekmann 1977; Diekmann et al. 1995).

Lastly, we would like to acknowledge the work of Forien et al. (2022), who consider a model very similar to ours (but without explicit vaccination), derive rigorously the scaling limit of the epidemic, and give criteria for the existence and asymptotic stability of an endemic equilibrium. We emphasize that, despite the striking similarities, the two models were formulated independently and most of the results presented in our work were obtained before that in Forien et al. (2022) were made available. Moreover, the main aim of our work is to draw public health insights from our model, letting sometimes mathematical rigour aside, whereas that of Forien et al. (2022) is mathematically much more accomplished and is targeted to an audience of probabilists. We believe that the two approaches offer complementary perspectives on the problem of waning immunity and endemicity.

The rest of this article is organized as follows. We start by describing our stochastic model and its large population size limit in Sect. 2. Then, in Sect. 3, we study the long-time behavior of the limiting equations and provide a simple criterion for the existence of an endemic equilibrium. In the following two sections, we examine the dependence of this criterion on the vaccination parameters to draw some public health insights from our model. We start with a general discussion in Sect. 4, and consider two more specific applications in Sect. 5, which require us to make a straightforward extension of our model to multiple groups. The well-posedness of the main PDE, introduced in Sect. 2, is proved in Sect. 6. Finally, a discussion on the model, the hypotheses and results is provided in Sect. 7.

## The model

### Model description

**Fig. 1:**
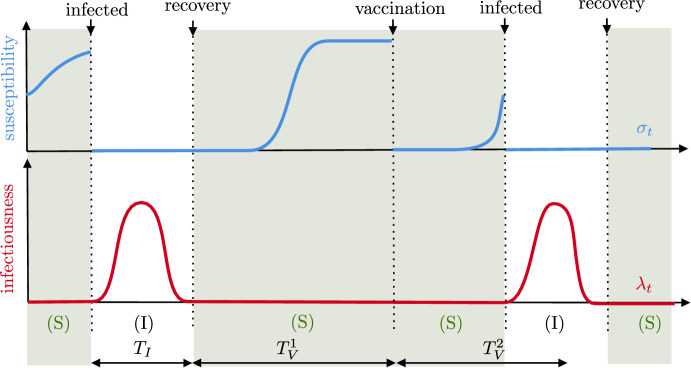
Typical evolution of the susceptibility $\sigma$ (in , on top) and infectiousness $\lambda$ (in , below) of an individual (color figure online)

**The dynamics without vaccination.** We consider the spread of a disease in a closed population of fixed size *N*, started at some reference time $t=0$ at which the state of the epidemic is known. Each individual in the population is characterized by two random quantities that change through time: its infectiousness, giving the rate at which it transmits the disease, and its susceptibility, corresponding to the probability that it gets reinfected upon contact with an infected individual. Individuals are labeled by $i\in \{1, \dots , N\}$, and the infectiousness of individual *i* at time *t* is denoted by $\lambda ^N_i(t)$ while its susceptibility is denoted by $\sigma ^N_i(t)$. We start by describing the dynamics of the epidemic in the absence of vaccination, and then indicate how vaccines are included to the model. The typical evolution of the susceptibility and infectiousness of an individual is represented in Fig. 1.

Consider a focal individual *i*. In the absence of vaccination, it goes repeatedly through two states. An infectious state (denoted by *I*), where it cannot be infected but can spread the disease ($\lambda ^N_i(t) \ge 0$ and $\sigma ^N_i(t) = 0$) and a susceptible state (denoted by *S*), where it does not spread the disease but can be reinfected ($\lambda ^N_i(t) = 0$ and $\sigma ^N_i(t) \ge 0$). The transition from *S* to *I* corresponds to the individual being infected and that from *I* to *S* to it recovering from the disease. Recovering might confer partial or even full immunity. As an *S* individual might be partially immune (if $\sigma ^N_i(t) < 1$), it is important to note that our definition of a susceptible individual is different from the usual one in differential equation models.

Upon infection, say at time $\tau$, individual *i* enters the *I* state and samples a random function $\lambda :[0, T_I) \rightarrow [0, \infty )$ according to a given distribution ${\mathcal {L}}_\lambda$. The length $T_I$ of the domain of $\lambda$ is random, and we think of it as being part of the definition of $\lambda$. The individual remains in the *I* state for a time period of length $T_I$, after which it *recovers* from the disease and moves to state *S*. During its infectious period, it cannot get reinfected, and its infectiousness is described by the function $\lambda$, that is,

$$\begin{aligned} \forall a < T_I,\quad \lambda ^N_i(\tau +a) = \lambda (a), \quad \sigma ^N_i(\tau +a) = 0. \end{aligned}$$

Once an individual has recovered from the disease, here at time $\tau '=\tau +T_I$, it cannot spread the disease anymore and acquires an immunity against reinfection that wanes. It enters the *S* state. To model that immunity is waning, the focal individual samples an independent random susceptibility $\sigma :[0, \infty ) \rightarrow [0, 1]$ according to another given distribution ${\mathcal {L}}_\sigma$ on the set of non-decreasing functions. We define

$$\begin{aligned} \forall a \ge 0,\quad \lambda ^N_i(\tau '+a) = 0, \quad \sigma ^N_i(\tau '+a) = \sigma (a). \end{aligned}$$

The susceptibility gives the probability to be infected upon exposure to the disease. More precisely, individual *i* gets reinfected at time *t* at rate $\sigma ^N_i(t)\Lambda ^N(t)$ with $\Lambda ^N$ the force of infection of the disease defined by

1 $$\begin{aligned} \forall t \ge 0,\quad \Lambda ^N(t) :=\frac{1}{N} \sum _{i=1}^N \lambda ^N_i(t). \end{aligned}$$

When such an event occurs, individual *i* goes back to the *I* state and we reproduce the above two steps independently. Note that since $\sigma ^N_i(t) = 0$ when *i* is in state *I*, only susceptible individuals can be reinfected. The interpretation of the latter expression is that each infectious individual, at a rate proportional to its infectiousness, makes a contact targeted to an individual chosen uniformly in the population. This contact leads to an infection with a probability given by the susceptibility of the target individual.

**Recovering the SIR, SIS, and SIRS models.** Let us rapidly illustrate how to recover the usual compartmental models from the previous definitions. Suppose that $\lambda$ and $\sigma$ are given by

$$\begin{aligned} \forall a < T_I,\quad \lambda (a) = \beta , \qquad \forall a \ge 0,\quad \sigma (a) = \textbf{1}_{\{ a \ge T_R\}} \end{aligned}$$

where $T_I$ is exponentially distributed with parameter $\gamma$, and $T_R$ is a random variable giving the length of the immune period. Then, infectious individuals yield new infections at a constant rate $\beta$, and stop being infectious (that is, recover) at rate $\gamma$. After having recovered, an individual is completely immune to reinfections ($\sigma (a) = 0$) for a random duration $T_R$, after which it becomes fully susceptible ($\sigma (a) = 1$). Depending on the distribution of $T_R$, we can recover either the SIR, SIS or SIRS model. If $T_R = \infty$ a.s., individuals are permanently immune following an infection and our model becomes a stochastic version of the SIR model. If $T_R = 0$ a.s., individuals build no immunity following an infection, and this corresponds to a stochastic SIS model. Finally, a stochastic SIRS model can be obtained by letting $T_R$ be exponentially distributed. Obviously, our model can account for much more complex situations, where infectiousness varies during the infectious period, and immunity is gradually lost following infection.

**Adding vaccines.** The previous rules describe the dynamics of the epidemic in the absence of vaccination. We model vaccines by assuming that vaccination has the same effect as (natural) immunization by the disease: upon vaccination the susceptibility of an individual is “reset” to a new independent random curve $\sigma '$ with law ${\mathcal {L}}_\sigma$, as if it had entered the *S* state after having recovered from the disease. We further assume that infectious individuals are not vaccinated, and that susceptible individuals are vaccinated recurrently according to a renewal process until they are reinfected. In particular, it might occur that an individual fully immune to the disease gets vaccinated.

More formally, if $\tau ''$ denotes a (random) time at which *i* is vaccinated, we set

$$\begin{aligned} \forall a \ge 0,\quad \lambda ^N_i(\tau ''+a) = 0, \quad \sigma ^N_i(\tau ''+a) = \sigma '(a), \end{aligned}$$

for an independent random variable $\sigma '$ with distribution ${\mathcal {L}}_\sigma$. After this event, a random independent duration $T_V$ is sampled according to some law ${\mathcal {L}}_V$, which gives the waiting time until individual *i* receives its next vaccine dose. If *i* has not been reinfected by time $\tau '' + T_V$, we reiterate the above two steps (resampling the susceptibility and a future vaccination time) independently. This process goes on until the individual is reinfected and goes back to the *I* state.

The dynamics of the population can be obtained by carrying out the previous steps altogether for each of the *N* individuals. Every time an individual is affected by an event (recovery, infection, vaccination), it samples its new susceptibility or infectiousness independently of all other individuals and of the past dynamics, according to ${\mathcal {L}}_\lambda$ in case of an infection or ${\mathcal {L}}_\sigma$ otherwise.

### Mathematical construction of the model

We now present a formal description of the model.

**Initial condition.** We suppose that the epidemic has been spreading for a long enough time that all individuals have been infected or vaccinated at least once at $t=0$. The initial condition we consider could easily be modified to encompass more general scenarios. We assume that a fraction $I_0$ of individuals are infected at $t=0$, and that $I_0 \in (0,1)$. We assign to each individual an initial state (*I* or *S*), age, susceptibility, and infectiousness independently in the following way. Consider a focal individual $i \in \{1,\dots , N\}$.

- We record the initial state of individual *i* as a random variable $C_{i,0} \in \{S, I\}$ such that
  $$\begin{aligned} \mathbb {P}(C_{i,0} = I) = 1 - \mathbb {P}(C_{i,0} = S) = I_0. \end{aligned}$$
- Individuals are assigned an initial age $A_i(0)=-\tau _{i,0}$ such that the age is distributed according to a probability density $h_I$ for an *I* individual and to a probability density $h_S$ for an *S* individual.

$$\circ $$
If individual *i* is in the *I* state: Conditional on $A_i(0)$, it is assigned an initial infectiousness $(\lambda _{i,0},T_{I,i,0})$ distributed as ${\mathcal {L}}_\lambda$ conditional on $T_I > -\tau _{i,0}$, so that the individual remains infectious at time $t=0$. Before time $T_{I,i,0}$, the age of *i* is $A^N_i(t) = A_i(0)+t$ and its infectiousness is
  $$\begin{aligned} \forall t < T_{I,i,0},\quad \lambda ^N_i(t) = \lambda _{i,0}(t+A_i(0)), \quad \sigma ^N_i(t) = 0. \end{aligned}$$
  After time $T_{I,i,0}$, individual *i* enters the *S* state and follows the dynamics described above.
$$\circ $$
If individual *i* is in the *S* state: Conditional on $A_i(0)$, it is assigned two independent variables: a susceptibility $\sigma _{i,0}$ distributed as ${\mathcal {L}}_V$ and an independent initial vaccination time $T_{V,i,0}$, with law ${\mathcal {L}}_V$ conditional on $T_V > A_i(0)$. Again, the age and susceptibility of *i* until time $T_{V,i,0}$ or until it gets infected are $A^N_i(t) = A_i(0) + t$ and
  $$\begin{aligned} \sigma ^N_i(t) = \sigma _{i,0}(t+A_i(0)). \end{aligned}$$

All these variables are assigned independently for different individuals.

**Spread of the epidemic.** Consider, for each *i*, three independent i.i.d. sequences, $(\lambda _{i,k}, T_{I,i,k};\, k \ge 1)$, $(\sigma _{i,k};\, k \ge 1)$, $(T_{V,i,k};\, k \ge 1)$ distributed as ${\mathcal {L}}_{\lambda }$, ${\mathcal {L}}_{\sigma }$ and ${\mathcal {L}}_V$. We also introduce for each *i* an auxiliary sequence of independent exponential random variables $(E_{i,k}; k \ge 1)$ with unit mean, independent of the previous sequences.

From these random variables and the initial condition, we will construct for each *i* two sequences of random variables $(\tau _{i,k};\, k \ge 1)$ and $(C_{i,k};\, k \ge 1)$ that represent respectively the time at which *i* experiences its *k*-th event (infection, recovery, or vaccination), and its state after this *k*-th event (*I* or *S*). Assuming that these variables are constructed, the age $A_i(t)$, the state $C_i(t)$, the infectiousness $\lambda _i^N(t)$ and the susceptibility $\sigma _i^N(t)$ of individual *i* at time *t* are simply given by

$$\begin{aligned} A_i(t)&=t-\tau _{i,K_i(t)},\quad C_i(t)=C_{i,K_i(t)}, \nonumber \\ \lambda _i^N(t)&= \lambda _{i,K_i(t)}\big (A_i(t)\big ) \textbf{1}_{\{C_i(t)=I\}}, \nonumber \\ \sigma _i^N(t)&= \sigma _{i,K_i(t)}\big (A_i(t)\big ) \textbf{1}_{\{C_i(t)=S\}}, \end{aligned}$$

where

$$\begin{aligned} \forall t \ge 0,\quad K_i(t) = \sup \{ k \ge 0: \tau _{i,k} < t \} \end{aligned}$$

is the number of events experienced by *i* at time *t*. We recall that the force of infection at time *t* is defined as

$$\begin{aligned} \Lambda ^N(t) = \frac{1}{N} \sum _{i=1}^N \lambda _i^N(t), \end{aligned}$$

with the convention that $\Lambda ^N \equiv 0$ for negative times.

Let us now construct $(\tau _{i,k};\, k \ge 1)$ and $(C_{i,k};\, k \ge 1)$ inductively. Suppose $\tau _{i,k}$ and $C_{i,k}$ have been constructed. We distinguish between two cases. If $C_{i,k} = I$, individual *i* eventually recovers so that we set $C_{i,k+1} = S$. This recovery occurs after a period of length $T_{I,i,k}$, and we define $\tau _{i,k+1} = \tau _{i,k} + T_{I,i,k}$. If $C_{i,k} = S$, the next event experienced by individual *i* is either a vaccination or a reinfection. We use $E_{i,k}$ to define the time of reinfection in the absence of vaccination as

2 $$\begin{aligned} Z_{i,k} = \inf \Big \{ a \ge 0: \int _0^a \Lambda ^N(\tau _{i,k}+u)\sigma _{i,k}(u) \textrm{d}u > E_{i,k} \Big \}. \end{aligned}$$

Note that $Z_{i,k}$ corresponds to the first atom of a Poisson point process with random intensity $\Lambda ^N(\tau _{i,k} + \cdot ) \sigma _{i,k}(\cdot )$. If $T_{V,i,k} > Z_{i,k}$, the individual gets reinfected before it is vaccinated. We set $C_{i,k+1} = I$ and $\tau _{i,k+1} = \tau _{i,k} + Z_{i,k}$. Otherwise, the individual is vaccinated before being reinfected, and we set $C_{i,k+1} = S$, and $\tau _{i,k+1} = \tau _{i,k} + T_{V,i,k}$.

**Fig. 2:**
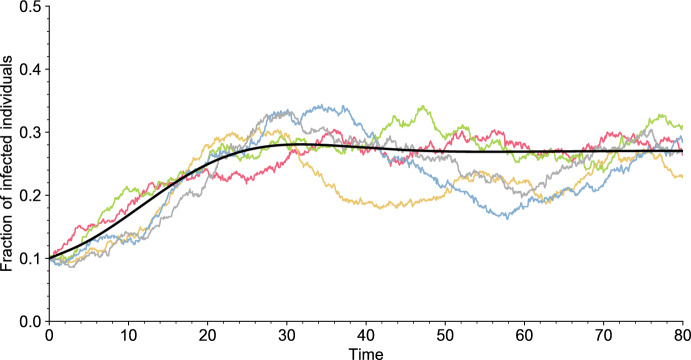
Independent simulations of the model (colored lines) for $N = 500$ and of its deterministic limit, as $N\rightarrow \infty$ (solid **black line**). All parameter values are given in Table 1 (Appendix A). The simulations are initialized with a fraction $I_0 = 0.1$ of infectious individuals (color figure online)

Overall, apart from the initial condition, the dynamics of the epidemic depends on four parameters: the population size *N*, the distribution of the infectiousness curve ${\mathcal {L}}_\lambda$, the distribution of the susceptibility curve ${\mathcal {L}}_\sigma$, and the distribution of the duration between two vaccinations ${\mathcal {L}}_V$. We will always assume that $T_I$ and $T_V$ have a density and a finite expectation. Some realizations of the model are displayed in Fig. 2.

**Population age structure.** Since the infectiousness and susceptibility are varying with time in our model, the state of the epidemic is not accurately described by counting the number of infectious and susceptible individuals. Deriving the large population size limit of the model requires to record for each individual a duration, which is the time elapsed since the last event (infection, recovery, or vaccination) that they experienced. We refer to this duration as the class age, or simply the age. Thus, the age of an *I* individual is the time elapsed since its infection, which is the classical definition of the age-of-infection in epidemic models (Thieme and Castillo-Chavez 1993; Inaba 2017). For an *S* individual it is the time since its last vaccination or recovery event, where we recall that an individual is said to recover when it moves from state *I* to state *S*. Recall that $A^N_i(t)$ is the age of individual *i* at time *t*, and $C^N_i(t) \in \{I, S\}$ its state. The relevant quantity that we will study is the age and state structure of the population, which we encode as the following two point measures on $[0, \infty )$

3 $$\begin{aligned} \forall t \ge 0,\quad \nu _I^N(t) = \frac{1}{N} \sum _{\begin{array}{c} i = 1,\dots ,N\\ C_{i}(t)=I \end{array}} \delta _{A^N_i(t)}, \quad \nu _S^N(t) = \frac{1}{N} \sum _{\begin{array}{c} i = 1,\dots ,N\\ C_{i}(t)=S \end{array}}\delta _{A^N_i(t)}. \end{aligned}$$

The measure $\nu ^N_I(t)$ (resp. $\nu ^N_S(t)$) has one atom for each infectious (resp. susceptible) individual at time *t*, with mass 1/*N* and whose location is the age of that individual.

### Large population size limit

The above stochastic model is too complicated to be studied directly. Instead, we will identify its large population size limit and use it as an approximation of the finite population model to study the efficiency of a vaccination strategy. We rely on a classical approach in statistical physics called *propagation of chaos* (sometimes also referred to as *molecular chaos*). The general idea is as follows: consider a stochastic system of *N* particles, initially independently distributed, with mean-field interaction between them. Focusing on a fixed number *k* of particles, under general assumptions the mean-field interaction averages out as the size of the system *N* tends to $\infty$, and the *k* particles behave independently in the limit. (The terminology propagation of chaos refers to the propagation in time of the initial independence of the particles when $N \rightarrow \infty$.) If the system is exchangeable, this asymptotic independence entails a law of large numbers for the empirical measure of the system. The limiting distribution usually has a density characterized as the unique solution of a nonlinear PDE. We refer the reader to Burkholder et al. (1989); Méléard (1996) for a detailed description of these concepts in the original context of kinetic theory, as well as to the recent exhaustive review by Chaintron and Diez (2022a, 2022b). For applications of these ideas in biological contexts, we refer to Chevallier (2017); Fournier and Löcherbach (2016) for models of interacting neurons, and finally to Forien et al. (2022) for a rigorous approach on a similar epidemiological model to ours.

We now apply these ideas to our model. The *N* particles correspond to the *N* individuals in the population, which interact in a mean-field way through infections. Since this approach is rather standard in the probabilistic literature, we only outline the usual arguments here. Recall the definition (3) of the empirical age distributions in the two compartments at time *t*, $\nu _I^N(t)$ and $\nu _S^N(t)$. We assume in this section that $\nu _I^N(t)$ (resp. $\nu _S^N(t)$) converges (in distribution as random measures) as $N \rightarrow \infty$ to a deterministic limiting measure with density $(I(t,a);\, a \ge 0)$ (resp. $(S(t,a);\, a \ge 0)$). We will identify the equations fulfilled by these limits. For any continuous bounded test function *f* we have

$$\begin{aligned} \int _0^\infty I(t,a)f(a)\textrm{d}a&= \lim _{N \rightarrow \infty } \mathbb {E}[\langle \nu _I^N(t), f\rangle ] \\&= \lim _{N \rightarrow \infty } \frac{1}{N} \sum _{i=1}^N \mathbb {E}[f(A_i^N(t)) \textbf{1}_{\{C_i^N(t)=I\}} ] \\&= \lim _{N \rightarrow \infty } \mathbb {E}[f(A_1^N(t)) \textbf{1}_{\{C_i^N(t)=I\}} ], \end{aligned}$$

where the last line follows from the exchangeability of the system and where we have used the standard notation $\langle \mu , f\rangle = \int f(x) \mu (\textrm{d}x)$. A similar computation holds for $\nu ^N_S(t)$. This shows that the limiting age structures of *I* and *S* individuals correspond to the limit in distribution of the age of one typical individual in the population, on the event that it is in state *I* or *S* respectively. Thus, we only need to understand the dynamics of a single individual, say individual $i=1$, in the limit $N \rightarrow \infty$.

According to the rules of the model, individual 1 only depends on the other individuals in the population through its infection rate $\sigma ^N_1(t) \Lambda ^N(t)$, with $\Lambda ^N$ given by (1). Assuming that $\Lambda ^N(t) \rightarrow \Lambda (t)$ as $N \rightarrow \infty$ for some deterministic function $\Lambda :=(\Lambda (t);\, t \ge 0)$, the limit in distribution of the age, state, infectiousness, and susceptibility of individual 1 is simply obtained by replacing $\Lambda ^N$ with its limit $\Lambda$ in the model description of Sect. 2.1. Using a similar notation as in Sect. 2.1, let us denote by $(\lambda ^\Lambda (t), \sigma ^\Lambda (t), A^\Lambda (t), C^\Lambda (t))$ the limit of the infectiouness, susceptibility, age and state of individual $i=1$ at time *t*, as $N \rightarrow \infty$. (That is, when $\Lambda ^N$ is replaced by $\Lambda$.) Now, by exchangeability as above

$$\begin{aligned} \Lambda (t)&= \lim _{N \rightarrow \infty } \mathbb {E}[\Lambda ^N(t)] = \lim _{N \rightarrow \infty } \frac{1}{N} \sum _{i=1}^N \mathbb {E}[\lambda ^N_i(t)]\\&= \lim _{N \rightarrow \infty } \mathbb {E}[\lambda ^N_1(t)] = \mathbb {E}[ \lambda ^\Lambda (t) ]. \end{aligned}$$

This puts the consistency constraint on $\Lambda$ that

4 $$\begin{aligned} \forall t \ge 0,\quad \Lambda (t) = \mathbb {E}[ \lambda ^\Lambda (t) ] \end{aligned}$$

should hold. In the terminology of propagation of chaos, a stochastic system satisfying (4) is called a solution to a McKean–Vlasov equation, or also a solution to a non-linear equation. It can be shown (see Proposition 1 in Sect. 6.1) that for our stochastic model, there exists a unique solution to the McKean–Vlasov equation (4). We denote it by $\Lambda ^*$, and by $(\lambda ^*(t), \sigma ^*(t),A^*(t), C^*(t))$ the corresponding quantities. The following result, that we state without proof, identifies the limit of the age and class structure of our model to the distribution of $(A^*(t), C^*(t))$ of the solution to the McKean–Vlasov equation. It has been proved in Forien et al. (2022) by making the above heuristic arguments rigorous.

#### Theorem 1

(Forien et al. 2022, Theorem 3.2) Suppose that there exists $\lambda _{\max }$ such that $\lambda (a) \le \lambda _{\max }$ almost surely for all $a \ge 0$. Then for any $t \ge 0$ we have

$$\begin{aligned} \lim _{N \rightarrow \infty } \nu ^N_I(t) = I(t,a) \textrm{d}a,\qquad \lim _{N \rightarrow \infty } \nu ^N_S(t) = S(t,a) \textrm{d}a \end{aligned}$$

in distribution for the topology of weak convergence. Furthermore, $(I(t,a);\, a \ge 0)$ is the density of $A^*(t)$ on the event $\{C^*(t) = I \}$, and $(S(t,a);\, a \ge 0)$ that on the event $\{C^*(t) = S \}$, where $(\lambda ^*(t), \sigma ^*(t), A^*(t), C^*(t);\, t \ge 0)$ is the (unique) solution to the above McKean–Vlasov Eq. (4).

### PDE formulation of the limit

The previous section has characterized the law of large numbers limit of the age structure of the epidemic in terms of the distribution of a stochastic system representing the limiting dynamics of a single individual in the population (of infinite size). We now provide a PDE formulation for this distribution, which can be thought of as the forward Kolmogorov equation associated to the previous stochastic process, although note that it is not a Markov process.

**Description of the limit.** Consider the following PDE, whose terms will be introduced throughout this section,

5 $$\begin{aligned} \begin{aligned} \partial _t I(t,a) + \partial _a I(t,a)&= - \mu _I(a) I(t,a) \\ \partial _t S(t,a) + \partial _a S(t,a)&= - \mu _V(a) S(t,a) - \Lambda (t) \mathbb {E}_{t,a}\big [ \sigma (a) \big ] S(t,a) \\ I(t,0)&= \Lambda (t) \int _0^\infty \mathbb {E}_{t,a}\big [ \sigma (a) \big ] S(t,a) \textrm{d}a\\ S(t,0)&= \int _0^\infty \mu _I(a) I(t,a) \textrm{d}a + \int _0^\infty \mu _V(a) S(t,a) \textrm{d}a \end{aligned} \end{aligned}$$

with initial conditions

$$\begin{aligned} I(0, a)&= I_0 h_I(a) \\ S(0, a)&= (1-I_0) h_S(a), \end{aligned}$$

where $I_0\in (0,1)$ is the fraction of infected individuals at $t=0$ and $h_I$ (resp. $h_S$) is age density of initially infectious (resp. susceptible) individuals.

Both the equation for *S* and *I* have a transport term corresponding to the aging phenomenon, and some removal terms corresponding to infections, vaccinations, and recoveries. Recovery (resp. vaccination) occurs at rate $\mu _I(a)$ (resp. $\mu _V(a)$) at age *a*, where $\mu _I(a)$ and $\mu _V(a)$ are the (age-dependent) recovery and vaccination rates respectively, which we assume to exist:

6 $$\begin{aligned} \forall a \ge 0,\quad \mu _I(a)\textrm{d}a = \frac{\mathbb {P}\big ( T_I \in [a,a+\textrm{d}a]\big )}{\mathbb {P}( T_I \ge a)}, \quad \mu _V(a) \textrm{d}a = \frac{\mathbb {P}\big ( T_V \in [a,a+\textrm{d}a]\big )}{\mathbb {P}( T_V \ge a)}.\nonumber \\ \end{aligned}$$

Newly recovered and vaccinated individuals become susceptible with age $a=0$ (typically implying being immune), yielding the two integrals in the age boundary condition for *S*.

The last and most interesting term corresponds to new infections. An individual is infected at a rate which is the product of its own susceptibility and of the force of infection in the population. In the limiting system, the force of infection is obtained by integrating the age-dependent infectiousness of *I* individuals over the age structure:

7 $$\begin{aligned} \forall t \ge 0,\quad \Lambda (t) = \int _0^\infty \mathbb {E}\big [\lambda (a) \mid T_I > a\big ] I(t,a) \textrm{d}a. \end{aligned}$$

We set $\Lambda \equiv 0$ for negative times. This is the usual expression for the force of infection in an epidemic model structured by time-since-infection (Kermack and McKendrick 1927; Diekmann et al. 1995; Brauer 2005). We define

8 $$\begin{aligned} \mathbb {E}_{t,a}\big [\sigma (a) \big ] = \mathbb {E}\Big [ \sigma (a) e^{-\int _0^a \Lambda (t-a+u) \sigma (u) \textrm{d}u} \Big ] \;\Big /\; \mathbb {E}\Big [ e^{-\int _0^a \Lambda (t-a+u) \sigma (u) \textrm{d}u} \Big ] \end{aligned}$$

to be the expected susceptibility of an *S* individual with age *a* at time *t*. The exponential term reflects that a susceptible individual with age *a* at time *t* is conditioned on not being infected between $t-a$ and *t*, which biases $\sigma$ in favor of a low susceptibility during this time period. This is an interesting example where the stochasticity of the underlying individual-based model does not entirely vanish in the large population size limit. Disregarding this stochasticity changes the limiting equations and hence the prediction of the model, even at the macroscopic scale. A similar conditioning is considered in Breda et al. (2012). Note that if $\sigma$ is deterministic the bias vanishes, that is, $\mathbb {E}_{t,a}[\sigma (a)] = \sigma (a)$. Our set of equations then becomes a version of the reinfection model of Kermack and McKendrick (Kermack and McKendrick 1932, 1933; Inaba 2001) in a closed population which incorporates vaccination. In our model, this amounts to discarding the inter-individuals variation in the immunity waning.

Finally we introduce the basic reproduction number $R_0$ as

9 $$\begin{aligned} R_0 = \int _0^{\infty }\mathbb {E}[\lambda (a)]\textrm{d}a, \end{aligned}$$

which we assume to be finite. As usual, $R_0$ represents the average number of secondary cases generated by an infected individual in a fully susceptible population.

**Weak solution and well-posedness.** We now introduce the definition of a weak solution to a general transport equations. Let *F* be a locally integrable function on $\mathbb {R}_+\times {\mathbb {R}}_+$. We say that $(f(t,a);\, t,a \ge 0)$ is a weak solution to

10 $$\begin{aligned} \partial _t f(t,a) + \partial _a f(t,a) = F(t,a) f(t,a) \end{aligned}$$

if

$$\begin{aligned} \forall a \le t,\quad f(t,a) = f(t-a, 0) \exp \Big ( \int _0^a F(t-a+u, u) \textrm{d}u\Big ) \\ \forall a \ge t,\quad f(t,a) = f(0, a-t) \exp \Big ( \int _{a-t}^a F(t-a+u, u) \textrm{d}u\Big ). \end{aligned}$$

This definition is motivated by a formal application of the method of characteristics. Suppose that *f* is a strong solution to the previous equation (in the sense that *f* is continuously differentiable and its partial derivatives verify (10) in the interior of the domain). We see that, along the characteristic line such $t-a$ is constant, *f* solves a first order linear differential equation. More precisely, by differentiating the map $g :u \mapsto f(t-u, a-u)$, it solves

$$\begin{aligned} g'(u) = -F(t-u, a-u) g(u). \end{aligned}$$

Solving this equation on $(0, t \wedge a)$ and noting that $g(0) = f(t,a)$ lead to the above expression. With this notion of weak solution, we can show that Eq. (5) is well-posed under mild technical assumptions.

#### Proposition 1

Equation (5) has a unique weak solution on the Skorokhod space ${\mathbb {D}}(\mathbb {R}^+,\mathbb {R}^+)$, when the following conditions hold

- there exists $\lambda _{\max }>0$ such that, $\forall a\ge 0$, $\mathbb {E}[\lambda (a)]\le \lambda _{\max }$,
- the density distribution functions of $T_I$ and $T_V$ and the functions
  11 $$\begin{aligned} &  t\mapsto \int _0^\infty \mu _I(t+a)\textrm{e}^{-\int _a^{a+t}\mu _I(u)\textrm{d}u}h_I(a)\textrm{d}a\nonumber \\ &  \quad +\int _0^\infty \mu _V(t+a)\textrm{e}^{-\int _a^{a+t}\mu _V(u)\textrm{d}u}h_S(a)\textrm{d}a \end{aligned}$$
  are bounded.

The above result is proved in Sect. 6.1. The next result connects the PDE (5) to the distribution of the solution to the McKean–Vlasov (4). It follows from elementary manipulations of point processes, and we postpone its proof until Sect. 6.2.

#### Proposition 2

Let $(\lambda ^*(t), \sigma ^*(t),A^*(t), C^*(t))$ be the solution to the McKean–Vlasov Eq. (4). Then, if $I(t, \cdot )$ (resp. $S(t, \cdot )$) is the density of $A^*(t)$ on the event $C^*(t) = I$ (resp. $C^*(t) = S$), $(I(t,a);\, t,a \ge 0)$ and $(S(t,a);\, t,a \ge 0)$ are the unique weak solutions to (5).

## Long-term behavior of the epidemic

### Equilibrium analysis

We are interested in the long-time behavior of Eq. (5). If this PDE converges to an equilibrium, the equilibrium should be a stationary solution of (5), that is, a solution which is independent of *t* and thus of the form

$$\begin{aligned} \forall t,a \ge 0,\quad I(t,a) = I(a),\quad S(t,a) = S(a). \end{aligned}$$

Similarly, let $\Lambda$ be the quantity defined in (7), but using the stationary age profile $(I(a);\, a \ge 0)$, and $\mathbb {E}_a[\sigma (a)]$ be defined through (8), using the stationary force of infection $\Lambda$. Note that these quantities no longer depend on the time variable. As is usual in similar epidemic models, we distinguish between two types of equilibria: disease-free equilibria, where there are no infected individuals in the population; and endemic equilibria, where the disease persists in the population.

**Disease-free equilibrium.** First, suppose that $I \equiv 0$. Then the PDE reduces to a first order linear differential equation,

$$\begin{aligned} \forall a \ge 0,\quad S'(a) = -\mu _V(a) S(a), \end{aligned}$$

whose unique solution is

12 $$\begin{aligned} \forall a \ge 0,\quad S(a) = S(0) \exp \Big ( -\int _0^a \mu _V(u) \textrm{d}u \Big ), \end{aligned}$$

where $S(0) = \mathbb {E}[T_V]^{-1}$ is so that $(S(a);\, a \ge 0)$ is a probability distribution. This shows that (5) always admits a unique disease-free equilibrium. Note that this equilibrium could have been easily anticipated. In the absence of infections, individuals only get vaccinated according to a renewal process with renewal time distribution $T_V$. Equation (12) is the stationary distribution of the time since the last vaccination event for this renewal process.

**Endemic equilibrium.** We now turn our attention to endemic equilibria ($I\not \equiv 0$). Let us make some computation to find an appropriate candidate. The two differential terms in (5) are reduced to the following linear differential equations for *I* and *S*:

$$\begin{aligned} \forall a \ge 0,\quad I'(a) = -\mu _I(a) I(a) \\ \forall a \ge 0,\quad S'(a) = -\mu _V(a) S(a) - \mathbb {E}_a[\sigma (a)] \Lambda S(a). \end{aligned}$$

Therefore any endemic equilibrium should fulfill that

13 $$\begin{aligned} \forall a \ge 0,\quad I(a) = I(0) \exp \Big (- \int _0^a \mu _I(u) \textrm{d}u\Big ) \end{aligned}$$

and

14 $$\begin{aligned} \forall a \ge 0,\quad S(a)&= S(0) \exp \left( - \int _0^a \mu _V(u) \textrm{d}u - \Lambda \int _0^a \mathbb {E}_u[\sigma (u)] \textrm{d}u\right) \nonumber \\&= S(0) \exp \left( - \int _0^a \mu _V(u) \textrm{d}u \right) \mathbb {E}\left[ e^{- \Lambda \int _0^a \sigma (u) \textrm{d}u } \right] . \end{aligned}$$

In the last line we have used that $g(a) = \mathbb {E}[ e^{- \Lambda \int _0^a \sigma (u) \textrm{d}u } ]$ is easily seen to solve $g'(a) =- \Lambda \mathbb {E}_a[\sigma (a)] g(a)$, where at equilibrium $\mathbb {E}_a[\sigma (a)]=\mathbb {E}[ \sigma (a) e^{-\Lambda \int _0^a \sigma (u) \textrm{d}u} ] \;\big / \; \mathbb {E}[ e^{-\Lambda \int _0^a \sigma (u) \textrm{d}u} ]$.

Recalling the definition of the force of infection (7), then using (13) and the definition of $R_0$ in (9),

$$\begin{aligned} \Lambda = \int _0^\infty I(a) \mathbb {E}[ \lambda (a) \mid T_I > a ] \textrm{d}a = I(0) \int _0^\infty \mathbb {E}[\lambda (a)] \textrm{d}a = I(0) R_0. \end{aligned}$$

Using the boundary condition for *I*, the fact that $\sigma$ and $T_V$ are independent, and the definition of $\mathbb {E}_a[\sigma (a)]$,

$$\begin{aligned} I(0)&= \Lambda \int _0^\infty \mathbb {E}_a[\sigma (a)] S(a)\textrm{d}a = S(0) \int _0^\infty e^{-\int _0^a \mu _V(u) \textrm{d}u} \mathbb {E}\Big [ \Lambda \sigma (a) e^{- \Lambda \int _0^a \sigma (u) \textrm{d}u } \Big ] \textrm{d}a \\&= S(0) \int _0^\infty \mathbb {E}\Big [ \textbf{1}_{\{T_V > a\}} \Lambda \sigma (a) e^{- \Lambda \int _0^a \sigma (u) \textrm{d}u } \Big ] \textrm{d}a = S(0) \mathbb {E}\Big [ 1 - e^{- \Lambda \int _0^{T_V} \sigma (u) \textrm{d}u } \Big ]. \end{aligned}$$

Therefore, an endemic equilibrium should verify that

15 $$\begin{aligned} S(0) = I(0) \,\Big /\, \mathbb {E}\Big [ 1 - e^{- R_0 I(0) \int _0^{T_V} \sigma (u) \textrm{d}u } \Big ]. \end{aligned}$$

Together, these computations lead to the following criterion for the existence of an endemic equilibrium.

#### Proposition 3

There exists an endemic equilibrium for each positive solution *x* of the equation $F_\textrm{e}(x) = R_0$ with

16 $$\begin{aligned} F_\textrm{e}(x) :=x \mathbb {E}[T_I] + x \frac{\displaystyle \mathbb {E}\Big [ \int _0^{T_V} e^{-x \int _0^a \sigma (u) \textrm{d}u} \textrm{d}a\Big ]}{\displaystyle \mathbb {E}\Big [1 - e^{-x \int _0^{T_V} \sigma (u) \textrm{d}u} \Big ]}. \end{aligned}$$

For a given solution *x*, the corresponding equilibrium is so that $I(0) = x / R_0$, *S*(0) is given by (15), and $(I(a);\, a \ge 0)$ and $(S(a);\, a \ge 0)$ by (13) and (14) respectively.

#### Proof

Suppose that $(I(a);\, a \ge 0)$ and $(S(a);\, a \ge 0)$ are a stationary solution of (5). Then, (14) shows that

$$\begin{aligned} \int _0^\infty S(a) \textrm{d}a&= S(0) \int _0^\infty \mathbb {P}(T_V > a) \mathbb {E}\Big [ e^{- R_0 I(0) \int _0^a \sigma (u) \textrm{d}u } \Big ] \textrm{d}a \\&= S(0) \mathbb {E}\Big [ \int _0^{T_V} e^{-R_0 I(0) \int _0^a \sigma (u) \textrm{d}u} \textrm{d}a \Big ]. \end{aligned}$$

Combining this to (13) and (15) yields

$$\begin{aligned} \int _0^\infty I(a) \textrm{d}a + \int _0^\infty S(a) \textrm{d}a = I(0) \mathbb {E}[T_I] + I(0) \frac{\displaystyle \mathbb {E}\Big [ \int _0^{T_V} e^{- R_0 I(0) \int _0^a \sigma (u) \textrm{d}u} \textrm{d}a\Big ]}{\displaystyle \mathbb {E}\Big [1 - e^{- R_0I(0) \int _0^{T_V} \sigma (u) \textrm{d}u} \Big ]} = 1 \end{aligned}$$

so that setting $x = I(0) R_0$ leads to a solution of (16).

Conversely, let *x* be a solution $F_\textrm{e}(x) = R_0$. Define $(I(a);\, a \ge 0)$ and $(S(a);\, a \ge 0)$ as in the statement of the result. The computation we have made already shows that both differential terms and the boundary condition for *I* are fulfilled. It is straightforward to check that the boundary condition for *S* is also fulfilled. All what remains to check is that

$$\begin{aligned} \int _0^\infty I(a) \textrm{d}a + \int _0^\infty S(a) \textrm{d}a = 1, \end{aligned}$$

which holds by making the same calculation as in the first part of the proof and using that *x* solves $F_\textrm{e}(x) = R_0$. $\square$

### The endemic threshold

From the characterization of the existence of endemic equilibria in the previous section, we see that there exists a threshold for $R_0$ under which there can be no endemic equilibrium. Indeed, since $F_\textrm{e}$ is continuous, we see that

$$\begin{aligned} \text {there exists an endemic equilibrium} \iff R_0 \ge \inf _{(\epsilon , \infty )} F_\textrm{e}(x)\, \text {for some}\, \epsilon > 0. \end{aligned}$$

We would like to obtain an explicit expression for this threshold and to study the uniqueness of an endemic equilibrium if it exists. This requires to study the variations of the function $F_\textrm{e}$. Let us start with two specific cases for which we can study the variations analytically. The two cases covered by this result are broad enough for many interesting applications, in particular choosing $\sigma$ to be of the form (17) leads to a stochastic version of the SIRS model, with general durations.

#### Proposition 4

Suppose that $\sigma$ is either deterministic or of the form

17 $$\begin{aligned} \forall a \ge 0,\quad \sigma (a) = \textbf{1}_{\{a \ge T_R\}} \end{aligned}$$

for some random duration $T_R$ with $\mathbb {E}[T_R] < \infty$. Then $F_\textrm{e}$ is increasing and there exists a unique endemic equilibrium if and only if $R_0 \Sigma > 1$ with

18 $$\begin{aligned} \Sigma :=\frac{\mathbb {E}\big [\int _0^{T_V} \sigma (a) \textrm{d}a\big ]}{\mathbb {E}[T_V]}. \end{aligned}$$

#### Proof

Define

$$\begin{aligned} \forall a \ge 0,\quad \phi (a) = \int _0^a \sigma (u) \textrm{d}u. \end{aligned}$$

The endemic function $F_\textrm{e}$ can be written as

19 $$\begin{aligned} \forall x > 0,\quad F_\textrm{e}(x) = x \mathbb {E}[T_I] + \dfrac{\mathbb {E}\Big [ \int _0^{T_V} e^{-x \phi (a)} \textrm{d}a\Big ]}{\mathbb {E}\Big [ \int _0^{T_V} \sigma (a)e^{-x \phi (a)} \textrm{d}a\Big ]}. \end{aligned}$$

We now distinguish between the two cases of the proposition.

*Step susceptibility.* If $\sigma$ is of the form $\sigma (a)=\textbf{1}_{\{a\ge T_R\}}$ for some random recovery duration $T_R$, we easily observe that

$$\begin{aligned} \mathbb {E}\Big [ \int _0^{T_V} e^{-x \phi (a)} \textrm{d}a\Big ]= &  \mathbb {E}[T_V\wedge T_R]+\mathbb {E}\left[ \textbf{1}_{\{T_V>T_R\}}\int ^{T_V}_{T_R}e^{-x (a-T_R)}\textrm{d}a\right] \\ \mathbb {E}\Big [ \int _0^{T_V} \sigma (a)e^{-x \phi (a)} \textrm{d}a\Big ]= &  \mathbb {E}\left[ \textbf{1}_{\{T_V>T_R\}}\int ^{T_V}_{T_R}e^{-x (a-T_R)}\textrm{d}a\right] . \end{aligned}$$

Consequently, $F_\textrm{e}(x)=x\mathbb {E}[T_I]+1+\mathbb {E}[T_V\wedge T_R]/\mathbb {E}[\int _0^{(T_V-T_R)_+}e^{-xa}\textrm{d}a]$, which is obviously an increasing function.

*Deterministic susceptibility.* From (19), we note that

$$\begin{aligned} F_\textrm{e}'(x) = \mathbb {E}[T_I] + \frac{h(x)}{\mathbb {E}\Big [ \int _0^{T_V} \sigma (a)e^{-x \phi (a)} \textrm{d}a\Big ]^2} \end{aligned}$$

with

$$\begin{aligned} h(x)= &  \mathbb {E}\left[ \int _0^{T_V} e^{-x \phi (a)} \textrm{d}a\right] \mathbb {E}\left[ \int _0^{T_V} \sigma (a)\phi (a)e^{-x \phi (a)} \textrm{d}a \right] \\ &  - \mathbb {E}\left[ \int _0^{T_V} \phi (a)e^{-x \phi (a)} \textrm{d}a\right] \mathbb {E}\left[ \int _0^{T_V} \sigma (a)e^{-x \phi (a)} \textrm{d}a\right] . \end{aligned}$$

Let $(T, \tilde{T})$ be a pair of independent copies of $T_V$. Since $\phi$ is deterministic

$$\begin{aligned} h(x)&= \mathbb {E}\left[ \int _0^T\int _0^{\tilde{T}} \sigma (b)\phi (b)e^{-x (\phi (a) + \phi (b))} \textrm{d}a \textrm{d}b\right] \\&- \mathbb {E}\left[ \int _0^T \int _0^{\tilde{T}} \phi (a) \sigma (b)e^{-x (\phi (a)+\phi (b))} \textrm{d}a \textrm{d}b \right] \\&=\mathbb {E}\left[ \int _0^T\int _0^{{\tilde{T}}} \left( \sigma (a)-\sigma (b)\right) \phi (a)e^{-x( \phi (a)+ \phi (b))} \textrm{d}a\textrm{d}b\right] \\&=\frac{1}{2}\mathbb {E}\left[ \int _0^T\int _0^{{\tilde{T}}} \left( \sigma (a)-\sigma (b)\right) \left( \phi (a)-\phi (b)\right) e^{-x( \phi (a)+ \phi (b))} \textrm{d}a\textrm{d}b\right] \ge 0, \end{aligned}$$

where we conclude using that $(\sigma (a)-\sigma (b))(\phi (a)-\phi (b)) \ge 0$ because both functions are non-decreasing. This shows that $F_\textrm{e}'(x) > 0$, proving that $F_\textrm{e}$ is increasing. $\square$

The critical value $1/\Sigma$ in (18) corresponds to the limit of $F_\textrm{e}$ at 0, which we can always compute (without any assumption on $\sigma$) as

$$\begin{aligned} \frac{1}{\Sigma } = \lim _{x \rightarrow 0} F_\textrm{e}(x) = \frac{\mathbb {E}[T_V]}{\mathbb {E}\big [\int _0^{T_V} \sigma (a) \textrm{d}a\big ]}. \end{aligned}$$

As a consequence, it is easily seen that in general there exists at least one endemic equilibrium if $R_0 \Sigma > 1$. However, having uniqueness of this equilibrium and absence of endemic equilibrium when $R_0\Sigma \le 1$ requires that $F_\textrm{e}$ is increasing, which we were not able to prove in general. Though, numerical simulations of $F_\textrm{e}$ suggest that it is an increasing function for a larger class of nondecreasing random curves $\sigma$, and we expect this to hold more generally, see Fig. 7 in Appendix A.1.

The criterion $R_0\Sigma > 1$ has an interesting interpretation in terms of the survival of a branching process (Athreya and Ney 1971). Writing

$$\begin{aligned} \Sigma = \frac{\mathbb {E}\big [ \int _0^{T_V} \sigma (a) \textrm{d}a \big ]}{\mathbb {E}[T_V]} = \int _0^\infty \mathbb {E}[\sigma (a)] S(a) \textrm{d}a, \end{aligned}$$

we note that $\Sigma$ corresponds to the mean susceptibility of the population at the disease-free equilibrium given by (12). Consider the epidemic generated by a single infected individual introduced in a population at the disease-free equilibrium. This individual makes on average $R_0$ infectious contacts with other individuals in the population over the course of its infection. The target of each such contact has a random susceptibility with expectation $\Sigma$, and thus the average number of infectious contacts actually leading to a new infection is $R_0 \Sigma$. As long as its size is small, the outbreak generated by the original infected individual can be approximated by a branching process with mean number of offspring $R_0 \Sigma$. This branching process can only lead to a large outbreak if it is supercritical, that is, if $R_0 \Sigma > 1$. Therefore, this criterion expresses that a vaccination policy prevents endemicity if it prevents a single infected individual in a population at the disease-free equilibrium from starting a large outbreak.

This interpretation of the threshold is reminiscent of the celebrated next-generation techniques in epidemic modeling (Diekmann et al. 1990, 2010) for assessing if a disease can invade a population with heterogeneous susceptibility, contacts, and infectiousness. However, note that in our model the susceptibility of an individual is not fixed, but changes as it gets vaccinated and its immunity is waning, and that the threshold characterizes the existence of an endemic equilibrium rather than the possibility of disease invasion. A similar interpretation was also proposed in Carlsson et al. (2020) for their model.

### Long-term behavior of the solutions

The computation in the previous section suggests that an endemic equilibrium exists if and only if $R_0 \Sigma > 1$. From a public health perspective, it is important to assess if this endemic equilibrium corresponds to the long-time behavior of our model when it exists. That is, we would like to assess the stability of this equilibrium.

In the simple case of the SIRS model (with no vaccination), when an endemic equilibrium exists (when $R_0 > 1$ in that case) it can be proved that it is globally asymptotically stable. In our more complicated setting, studying mathematically the stability of the endemic equilibrium seems out of reach. We thus investigate this question numerically.

**Fig. 3:**
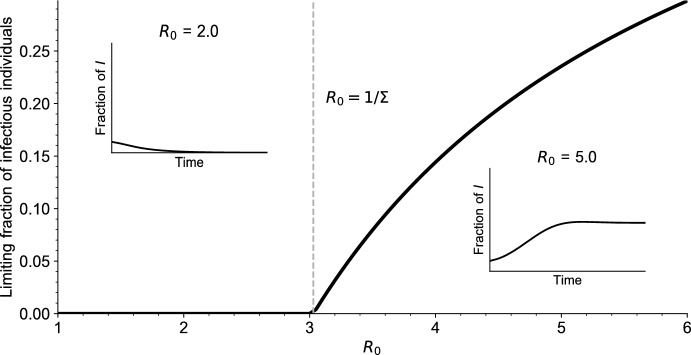
Bifurcation diagram for Eq. (5). For each value of $R_0$, the value of $\int _0^\infty I(t,a) \textrm{d}a$ is reported, for a large time $t = 5000$. The simulations are initialized with a fraction $I_0 = 0.1$ of infectious individuals, all other parameter values are given in Table 1 (Appendix A). The dashed vertical grey line indicates the endemic threshold $1/\Sigma$ computed from (18), above which we expect to see existence of a stable endemic equilibrium. In the two insets $\int _0^\infty I(s,a) \textrm{d}a$ is plotted as a function of time $s\le t$ for $R_0 = 2$ and $R_0 = 5$ (color figure online)

**Fig. 4:**
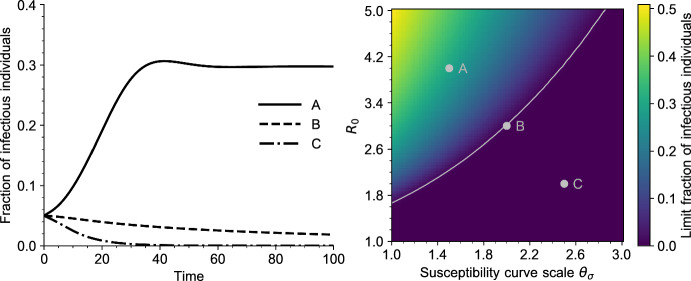
Left: Solutions of the PDE (5) for three values of $R_0$ and $\theta _\sigma$. The parameters correspond to the grey dots on the right plot. All other parameters are given in Table 1 (Appendix A). Right: Bifurcation diagram of Eq. (5), as a function of $R_0$ and $\theta _\sigma$ (scale parameter, defined in Sect. A.1). Each point of the heatmap represents the value of $\int _0^\infty I(t,a) \textrm{d}a$ for a large time $t = 300$. The grey curve is the endemic threshold $1/\Sigma$ defined in (18), as a function of $\theta _\sigma$ (color figure online)

In Figs. 3 and 4 we draw the bifurcation diagram of our system, as well as some typical trajectories of the fraction of infected individuals in our model. A first observation is that, on the simulation displayed in Fig. 3 and in all simulations carried out by the authors, the model exhibits a simple asymptotic behavior. The epidemic either dies out, in the sense that the fraction of infected individuals goes to 0, or survives in which case the fraction of infected individuals converges to a positive value. Moreover, the region of parameters for which the epidemic survives coincides with the region of parameters for which $R_0 \Sigma > 1$, that is, for which we predict the existence of an endemic equilibrium.

Overall, this suggests that the asymptotic behavior of our model is very similar to that of the more usual systems of ordinary differential equations of the SIRS type: when an endemic equilibrium exists, it is globally asymptotically stable, otherwise the disease-free equilibrium is globally asymptotically stable. When $R_0$ crosses the threshold $1/\Sigma$, we observe an exchange of stability of the two equilibria, similar to a transcritical bifurcation.

Let us make a final remark. The solution to the PDE (5) has a probabilistic interpretation as the age distribution of the solution to the McKean–Vlasov Eq. (4). In this probabilistic setting, existence of an endemic equilibrium translates into existence of a stationary age distribution, and proving the asymptotic stability of this equilibrium amounts to proving convergence of the age distribution towards the stationary distribution. This connexion could provide a way to study the stability of endemic equilibria analytically. We refer to models using piecewise deterministic Markov processes with age dependence such as Bouguet (2015); Fournier and Löcherbach (2016) for similar ideas.

## Impact of the vaccination policy on endemicity

In the light of the results of the previous section, the long-term behavior of the epidemic depends mostly on three parameters, namely $R_0$, ${\mathcal {L}}_\sigma$ and ${\mathcal {L}}_V$ (the distributions of $\sigma$ and $T_V$). In this section, we discuss the impact that policy-making can have on the control of the epidemic through changing these parameters.

Policy-making does not impact these three parameters in the same way. The basic reproduction number $R_0$ can be lowered by reducing the contact rate in the population, but is not dependent on the way vaccines are administrated. We will consider it as fixed since we are mostly interested in studying the impact of vaccination rather than changes in the contact rate. Similarly, we think of $\sigma$ as reflecting the protection against reinfection provided by the host immunity. The waning of this protection is therefore dictated by the biological features of the disease and of the host immunity, which cannot be influenced by policy-making. (We do neglect the fact that part of the variation of the susceptibility might come from behavioral changes that could be affected by policy.) We thus consider the law ${\mathcal {L}}_\sigma$ of $\sigma$ as being also fixed. Finally, we think of $T_V$ as resulting from the vaccination strategy being applied. Typically, the law of $T_V$ depends on the number of doses administrated, on the instructions given to the general population on when and how often to get vaccinated, and on how these instructions are being followed. The law of $T_V$ has a complicated effect on the outcome of the disease, which depends strongly on the distribution of $\sigma$ and that we aim to study.

In the rest of this work, we will use $\Sigma$ as an indicator of the efficiency of the vaccination policy, and try to see what distribution ${\mathcal {L}}_V$ of $T_V$ might achieve a lower $\Sigma$.

### The cost of a vaccination policy

Intuitively, vaccinating the population more often on average should result in a higher protection against transmissions, but comes at a higher cost (of producing the vaccines and deploying them for instance). We will quantify this cost in order to compare the efficiency of a vaccination strategy (that is, of a distribution of $T_V$) relative to its cost, and not only in absolute terms.

A natural measure of the cost of a vaccination policy is the per capita per unit of time number of vaccine doses that are injected. In our model, the number of doses injected between time *t* and $t + \textrm{d}t$ is

$$\begin{aligned} \int _0^\infty S(t,a) \mu _V(a) \textrm{d}a \cdot \textrm{d}t. \end{aligned}$$

If the population is at the disease-free equilibrium (12), a simple computation shows that the number of doses administrated per unit of time is

$$\begin{aligned} \int _0^\infty S(a) \mu _V(a) \textrm{d}a = \frac{1}{\mathbb {E}[T_V]}. \end{aligned}$$

We argue that, as long as the incidence and prevalence of the disease are low, the number of vaccines doses used per unit of time at the endemic equilibrium can also be approximated by $1/\mathbb {E}[T_V]$.

Let us suppose that the population is at an endemic equilibrium, and that the incidence is negligible, that is, that $I(0) \ll 1$. Using (13) and the latter assumption on the incidence,

$$\begin{aligned} \int _0^\infty I(a) \textrm{d}a = I(0) \mathbb {E}[T_I] \ll 1 \end{aligned}$$

so that the prevalence of the disease should also be low. From (14) and using $I(0) \ll 1$ we compute that the number of doses injected per unit of time at the endemic equilibrium is approximated by

$$\begin{aligned} \int _0^\infty S(0) \mu _V(a) \exp \left( - \int _0^a \mu _V(u) \textrm{d}u \right) \mathbb {E}\left[ e^{- I(0) R_0 \int _0^a \sigma (u) \textrm{d}u } \right] \textrm{d}a \approx S(0). \end{aligned}$$

Using that the prevalence is negligible, we further deduce from

$$\begin{aligned} 1 = \int _0^\infty S(a) \textrm{d}a + \int _0^\infty I(a) \textrm{d}a \approx \int _0^\infty S(0) \exp \left( - \int _0^a \mu _V(u) \textrm{d}u \right) \textrm{d}a = S(0) \mathbb {E}[T_V] \end{aligned}$$

that the number of doses injected can be approximated by $S(0) \approx 1/\mathbb {E}[T_V]$.

Overall, based on this heuristic computation, we will use $1/\mathbb {E}[T_V]$ as an indicator of the cost of a vaccination strategy $T_V$.

### Impact of the vaccination strategy

We now study the effect that modifying the distribution of $T_V$ has on the value of $\Sigma$ defined in (18). By using Fubini’s theorem, let us first re-write the expression for $\Sigma$ as

$$\begin{aligned} \Sigma = \frac{\mathbb {E}\big [\int _0^{T_V} \sigma (a) \textrm{d}a \big ]}{\mathbb {E}[T_V]} = \frac{\mathbb {E}[\Phi (T_V)]}{\mathbb {E}[T_V]}, \end{aligned}$$

where the deterministic function $\Phi$ is defined as

$$\begin{aligned} \forall t \ge 0,\quad \Phi (t) = \mathbb {E}\Big [ \int _0^t \sigma (a) \textrm{d}a \Big ] = \int _0^t \mathbb {E}[\sigma (a)] \textrm{d}a. \end{aligned}$$

**Optimal strategy for a fixed cost.** We assume that only a fixed number of doses can be administrated per unit of time in the population, say 1/*m*, so that we restrict our attention to random variables $T_V$ verifying $\mathbb {E}[T_V] = m$. What distribution of $T_V$ then achieves the smallest value of $\Sigma$? In other words, given that a fixed daily number of doses are available, how are these doses best distributed to achieve the highest average immunity level in the population?

It turns out that this question is easily answered analytically. Since $a \mapsto \sigma (a)$ is a.s. nondecreasing, the function $a \mapsto \Phi (a)$ is convex. Therefore, applying Jensen’s inequality we obtain that

$$\begin{aligned} \Sigma = \frac{\mathbb {E}[\Phi (T_V)]}{\mathbb {E}[T_V]} \ge \frac{\Phi (m)}{m}, \end{aligned}$$

where we recall that we have assumed that $\mathbb {E}[T_V] = m$. We see that the right-hand side of the previous inequality, $\Phi (m)/m$, is the susceptibility at the disease-free equilibrium when $T_V = m$ almost surely. It corresponds to an idealized situation where each individual gets vaccinated every *m* unit of time, exactly. Therefore, given a vaccination strategy $T_V$, a better strategy that uses the same number of doses is always to let each individual receive vaccines at evenly spaced moments.

The optimal allocation strategy with $\mathbb {E}[T_V] = m$ is achieved by letting $T_V = m$ a.s., that is, by letting $T_V$ follow the distribution with the smallest dispersion. More generally, we argue that a distribution of $T_V$ which is less dispersed performs better at preventing an endemic state. Intuitively, if $T_1$ is less dispersed than $T_2$ and both have the same mean, the distribution of $T_2$ has more mass at larger times. Loosely speaking, the contribution of large values to the integral of a convex function is large, and the value of $\mathbb {E}[\Phi (T_2)]$ should be larger. Being more rigorous, one common way to formalize the notion of dispersal is to use the notion of *convex ordering* (Shaked and Shanthikumar 2007, Cha. 3). A random variable $T_1$ is smaller in convex ordering than another random variable $T_2$ if

$$\begin{aligned} \forall \phi \text { convex},\quad \mathbb {E}[\phi (T_1)] \le \mathbb {E}[\phi (T_2)], \end{aligned}$$

which we write as $T_1 \preceq T_2$. Being larger in convex ordering is a common indicator of larger dispersion. Trivially, if $T_1 \preceq T_2$ and $\Sigma _1$ and $\Sigma _2$ are the respective stationary susceptibilities, we have that $\Sigma _1 \le \Sigma _2$. Overall, this indicates that larger variability in the vaccination times perform worse at preventing the spread of the disease at the population level.

Note that vaccinating at a fixed time after recovery was also found to be the optimal vaccination strategy by El Khalifi and Britton (2023) for a related model. Although, the modeling of the vaccination strategies is different in this article and in our work.

**Effect of increasing the vaccination effort.** We now consider the effect of varying the mean of $T_V$, which can be interpreted as varying the number of vaccine doses administrated in the population. Intuitively, one would expect that reducing $\mathbb {E}[T_V]$ (that is, vaccinating more) also reduces the stationary susceptibility $\Sigma$ (that is, leads to a higher level of immunity). However, it is not hard to come up with counter-examples where this is not the case, and no conclusion can be drawn in general.

Nonetheless, it is a reasonable assumption to suppose that increasing the number of vaccines administrated will not drastically change the shape of the distribution of $T_V$, but rather modify it in a continuous way. One way to model this effect is to consider a variable $m \ge 0$ representing the (inverse of the) vaccination effort. At vaccination effort *m*, the vaccination period is distributed as $mT_V$, for some fixed random variable $T_V$. (Increasing the number of vaccines only changes the *scale* of the distribution.) Clearly, since $\Phi$ is convex, the function

$$\begin{aligned} m \mapsto \frac{\mathbb {E}[ \Phi (m T_V) ]}{\mathbb {E}[mT_V]} \end{aligned}$$

is increasing. We do recover the expected and intuitive behavior: as *m* increases, less vaccines are administrated, and $\Sigma$ increases, that is, the population becomes more susceptible to the disease.

## Public health applications

As an application of our model, we now study in more details two specific situations. In the first situation, we assume that a fraction of the population does not get vaccinated. This could reflect among other examples vaccine hesitancy, impossibility to receive a vaccine, or unequal access to the vaccination. We want to understand the impact at the population level of having such a subpopulation that is not vaccinated, and to derive an expression for the minimal fraction of the population that needs to be vaccinated to prevent endemicity.

In the second situation, we consider that the population is divided into two groups, which can represent two distinct physical locations (cities, countries), or two groups in a heterogeneous population. We assume that a fixed number of vaccine doses can be administrated per unit of time, due to resource limitation such as limited vaccine production or deployment. We investigate the impact of an uneven allocation of these doses between the two groups.

In both situations the population is no longer homogeneous, in the sense that it is made of several groups with a different vaccination policy enforced in each group. We start by making a straightforward extension of our model to such a heterogeneous population in Sect. 5.1. We will derive briefly in Sect. 5.2 a corresponding law of large numbers and criterion for the existence of an endemic equilibrium, similar to that for the homogeneous model. In Sect. 5.3 we provide some general results in the case of two subpopulations, and we finally our two situations of interest in Sect. 5.4.

### Modeling vaccination heterogeneity

In this section, we consider a population of size *N* divided into *L* subgroups. These groups model some heterogeneity in the population such as age classes, physical locations, or compliance to public health recommendations. We suppose that these groups mix heterogeneously according to some contact matrix, modeling for example heterogeneous social mixing (Prem et al. 2017; Koltai et al. 2022) or mobility patterns between different physical areas (Balcan et al. 2009; Merler and Ajelli 2010). We also assume that different groups are not vaccinated at the same rate, modeling for instance vaccination policies targeted at specific groups (Hardt et al. 2016), heterogeneous administration of vaccines (Perry et al. 2021; Mathieu et al. 2021), or differences in beliefs and compliance to public health recommendations (Hofmann et al. 2006; Downs et al. 2008; Lazarus et al. 2023). We make the simplifying assumption that the group to which an individual belongs does not change during the course of the epidemic, and that the distribution of the susceptibility and infectiousness curves do not depend on the group. Let us give a more precise definition of the dynamics and of the parameters of this extension.

**Description.** Suppose that each of the *N* individuals in the population now belongs to one of *L* groups, labeled by $\ell \in \{ 1, \dots , L \}$. We assume that individuals remain in the same group at all times. The number of individuals in group $\ell$ is denoted by $N_\ell$, and we assume that $N_\ell / N \rightarrow p_\ell \in (0, 1)$ as $N \rightarrow \infty$. An individual is now identified by a pair $(\ell , i)$, $\ell$ being its group and $i \le N_\ell$ its label within group $\ell$. We will denote its infectiousness and susceptibility at time *t* by $\lambda ^N_{\ell ,i}(t)$ and $\sigma ^N_{\ell ,i}(t)$ respectively. Individuals follow the same dynamics as described in Sect. 2.1 with two modifications: contacts are heterogeneous between groups and the group of an individual affects its vaccination rate.

Heterogeneity in the contacts is encoded as a symmetric matrix $\Gamma =(\gamma _{\ell , \ell '})$, where $\gamma _{\ell ,\ell '} = \gamma _{\ell ',\ell } \ge 0$ gives the contact intensity between an individual of group $\ell$ and one of group $\ell '$. We assume that this contact matrix does not depend on the size of the population *N*. Note that $\gamma _{\ell , \ell '}$ corresponds to a contact rate per pair of individuals, so that the overall contact rate between group $\ell$ and group $\ell '$ is $\gamma _{\ell , \ell '} N_\ell N_{\ell '}$. The rate at which individual $(\ell ,i)$ gets infected at time *t* is

$$\begin{aligned} \sigma ^N_{\ell ,i}(t) \cdot \sum _{\ell ' = 1}^L \gamma _{\ell ',\ell } \Lambda ^N_{\ell '}(t), \end{aligned}$$

where

$$\begin{aligned} \Lambda ^N_\ell (t) = \frac{1}{N} \sum _{i'=1}^{N_\ell } \lambda ^N_{\ell , i'}(t). \end{aligned}$$

In words, each individual $(\ell ', i')$ makes an infectious contact with $(\ell , i)$ at rate $\gamma _{\ell ',\ell } \lambda ^N_{\ell ',i'}(t) / N$, and such an infectious contact at time *t* yields an infection with probability $\sigma ^N_{\ell ,i}(t)$.

Upon infection, an individual samples an infectious period $T_I$ and an infectiousness $\lambda$ according to the same distribution ${\mathcal {L}}_\lambda$ as in the homogeneous model, regardless of its group. Upon entry in the *S* state, the susceptibility $\sigma$ of any individual is also sampled according to the same common distribution ${\mathcal {L}}_{\sigma }$ which does not depend on the group. However, an individual in group $\ell$ samples its waiting time until the next vaccination according to the distribution of a random variable $T_\ell$ that depends on the group. (Note that we have dropped the *V* subscript to ease the notation.)

This model could be easily made more general by allowing the distribution of the infectiousness and susceptibility ${\mathcal {L}}_\lambda$ and ${\mathcal {L}}_\sigma$ to depend on the group. This could represent a heterogeneous vulnerability to the disease for instance.

**Large population size limit.** As in the homogeneous model, we can derive a law of large numbers limit for the age and state structure of the epidemic. Let the empirical measure of ages of *I* and *S* individuals in group $\ell$ be denoted respectively as

$$\begin{aligned} \nu _{I,\ell }^N(t)= &  \frac{1}{N} \sum _{\begin{array}{c} i = 1,\dots ,N_\ell \\ C^N_{\ell ,i}(t)=I \end{array}} \delta _{A^N_{\ell ,i}(t)},\\ \nu _{S,\ell }^N(t)= &  \frac{1}{N} \sum _{\begin{array}{c} i = 1,\dots ,N_\ell \\ C^N_{\ell ,i}(t)=S \end{array}} \delta _{A^N_{\ell ,i}(t)}. \end{aligned}$$

If the initial age structures converge, the above empirical measures should converge respectively, as $N \rightarrow \infty$, to the solution $(I_\ell (t,a);\, a \ge 0)$ and $(S_\ell (t,a);\, a \ge 0)$ of the following multidimensional version of Eq. (5),

20 $$\begin{aligned} \begin{aligned} \partial _t I_\ell (t,a) + \partial _a I_\ell (t,a)&= - \mu _I(a) I_\ell (t,a) \\ \partial _t S_\ell (t,a) + \partial _a S_\ell (t,a)&= - \mu _{V,\ell }(a) S_\ell (t,a) - \sum _{\ell '=1}^L \gamma _{\ell ',\ell } \Lambda _{\ell '}(t) \cdot \mathbb {E}_{t,a;\ell }[\sigma (a)] S_\ell (t,a) \\ I_\ell (t,0)&= \sum _{\ell ' = 1}^L \gamma _{\ell ',\ell } \Lambda _{\ell '}(t) \cdot \int _0^\infty \mathbb {E}_{t,a;\ell }[\sigma (a)] S_\ell (t,a) \textrm{d}a \\ S_\ell (t,0)&= \int _0^\infty \mu _{V,\ell }(a) S_\ell (t,a) \textrm{d}a + \int _0^\infty \mu _I(a) I_\ell (t,a) \textrm{d}a. \end{aligned} \end{aligned}$$

The initial condition of this system of PDE is a straightforward extension of that in Eq. (5) and we do not write it down explicitly. Nevertheless note that the initial condition should fulfill that

$$\begin{aligned} \forall \ell \le L,\quad \int _0^\infty I_\ell (0,a) + S_\ell (0,a) \textrm{d}a = p_\ell , \end{aligned}$$

where $$p_\ell =\lim _{N\rightarrow +\infty }{N_\ell }/{N}.$$

In the previous equation, $\mu _{V,\ell }(a)$ denotes the vaccination rate in group $\ell$, obtained by replacing $T_V$ by $T_{\ell }$ in (6), and we define

$$\begin{aligned} \Lambda _{\ell }(t) = \int _0^\infty I_\ell (t,a) \mathbb {E}[ \lambda (a) \mid T_I > a] \textrm{d}a \end{aligned}$$

and

$$\begin{aligned} \mathbb {E}_{t,a;\ell }[\sigma (a)]= &  \mathbb {E}\Big [ \sigma (a) \exp \Big (- \int _0^a \sum _{\ell '=1}^L \gamma _{\ell ',\ell } \Lambda _{\ell '}(t-a+u) \sigma (u) \textrm{d}u\Big ) \Big ] \\ &  \Big /\, \mathbb {E}\Big [ \exp \Big (- \int _0^a \sum _{\ell '=1}^L \gamma _{\ell ',\ell } \Lambda _{\ell '}(t-a+u) \sigma (u) \textrm{d}u\Big ) \Big ]. \end{aligned}$$

All other terms have been defined in Sect. 2.4.

### Endemicity criterion for heterogeneous vaccination

Again, we study the equilibria of (20) to derive a criterion for the existence of an endemic equilibrium. We look for solutions of (20) of the form

$$\begin{aligned} \forall t, a \ge 0,\, \forall \ell \in \{1, \dots , L\},\quad I_\ell (t,a) = I_\ell (a),\quad S_\ell (t,a) = S_\ell (a). \end{aligned}$$

We will assume from now on that the matrix $\Gamma$ is irreducible. In this case, it is not hard to see that there are only two possible types of equilibria: either the disease is absent in each groups ($I_\ell \equiv 0$ for all $\ell \in \{1, \dots , L\}$) or it is endemic in each group ($I_\ell > 0$ for all $\ell \in \{ 1, \dots , L\}$). Naturally, we will refer to the former situation as a disease-free equilibrium, and to the latter one as an endemic equilibrium.

**Disease-free equilibrium.** As in the homogeneous case, in the absence of infected individuals the only remaining dynamics are the vaccination according to renewal processes. It is not hard to check that the only equilibrium of (20) with $I_\ell (a) \equiv 0$ for all $\ell \ge 1$ is given by

$$\begin{aligned} \forall a \ge 0,\, \forall \ell \in \{1, \dots , L\},\quad S_\ell (a) = \frac{p_\ell }{\mathbb {E}[T_\ell ]} \exp \Big (-\int _0^a \mu _{V,\ell }(u) \textrm{d}u \Big ). \end{aligned}$$

**Endemic equilibrium.** In principle, we could use the same arguments as in the homogeneous case and find a set of *L* coupled equations similar to (16) that characterize the existence of stationary points of (20). However, solving these equations would prove to be an even more difficult task in this multi-dimensional setting. We choose not to go in this direction and prefer to start from the connection between the endemicity criterion in the homogeneous case and the survival of a well-chosen branching process.

Suppose that a single individual of group $\ell$ is infected in a population at the disease-free equilibrium. Over its entire infectious period, this individual makes on average $p_{\ell '} \gamma _{\ell , \ell '} R_0$ infectious contacts with individuals of type $\ell '$. An individual of group $\ell '$ targeted by an infectious contact has a random susceptibility. The expectation of this random variable is the mean susceptibility at the disease-free equilibrium of group $\ell '$, that is,

21 $$\begin{aligned} \Sigma _{\ell '} :=\frac{1}{p_{\ell '}} \int _0^\infty S_{\ell '}(a) \mathbb {E}[\sigma (a)] \textrm{d}a = \frac{\mathbb {E}\big [ \int _0^{T_{\ell '}} \sigma (a) \textrm{d}a\big ]}{\mathbb {E}[T_{\ell '}]}. \end{aligned}$$

Therefore, an infected individual of group $\ell$ produces on average $R_0 m_{\ell ,\ell '}$ secondary infections in group $\ell '$, with

22 $$\begin{aligned} m_{\ell ,\ell '}:= p_{\ell '} \gamma _{\ell , \ell '} \Sigma _{\ell '}. \end{aligned}$$

We introduce the matrix

$$\begin{aligned} M :=(m_{\ell ,\ell '})_{1\le \ell ,\ell '\le L}. \end{aligned}$$

According to the previous discussion, the epidemic generated by a single infected individual can be thought of as a multi-type branching process with mean offspring matrix $R_0 M$. The type of an individual in the branching process corresponds to the group to which it belongs. It is now a classical result from the theory of branching processes that, under our mild condition that the contact matrix $\Gamma$ is irreducible, the latter branching process can survive with positive probability if and only if the leading eigenvalue of its mean offspring matrix $R_0 M$ is larger than 1, that is, if and only if $R_0 \rho > 1$, where $\rho$ is the leading eigenvalue of the matrix *M* (Athreya and Ney 1971, Chapter V). This is again reminiscent of the next-generation matrix techniques of Diekmann et al. (1990, 2010).

**Fig. 5:**
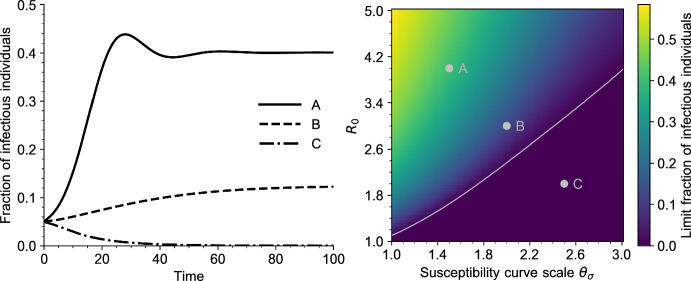
Left: Solutions of the PDE (5) for three values of $R_0$ and $\theta _\sigma$. The parameters correspond to the grey dots on the right plot. All other parameters are given in Table 1 (Appendix A). Right: Bifurcation diagram of Eq. (20), as a function of $R_0$ and $\theta _\sigma$ (scale parameter, defined in Sect. A.1). The population is made of three subpopulations with contact matrix and vaccination parameters given in Table 2. Each point of the heatmap represents the value of the total fraction of infectious individuals $\int _0^\infty I_1(t,a)+I_2(t,a)+I_3(t,a) \textrm{d}a$ for a large time $t = 300$. The grey curve is the endemic threshold $1/\rho$ as a function of $\theta _\sigma$, where $\rho$ is the leanding eigenvalue of *M* defined in (22) (color figure online)

**Asymptotic behavior.** As in the case of homogeneous contacts, we are ultimately interested in assessing the effect of the parameters of the model on the persistence of the disease in the population for a long time. The criterion that we have derived above for the existence of an endemic equilibrium is heuristic, and does not guarantee that the state of the population converges to that endemic equilibrium when it exists.

Again, we study the asymptotic behavior of the PDE numerically by considering the bifurcation diagram of our model in Fig. 5, in the case of three subpopulations. The trajectory of the total fraction of infected individuals among all groups is plotted for a sample of typical trajectories. As in the homogeneous case, the asymptotic behavior of the model is simple, and it seems to converge to a limit. In this limit, depending on the parameters, the epidemic is either extinct or has reached an endemic equilibrium. Again, we see a good agreement between our theoretical prediction for the existence of an endemic equilibrium ($R_0\rho > 1$) and the parameter region where the epidemic does not go extinct. This also validates that our heuristic, based on the survival probability of a certain multi-type branching process, seems to give the right criterion for the existence of an endemic equilibrium.

### General results on two groups

We will discuss our two applications in the simpler context of only two subpopulations, $L = 2$. Before considering these applications, let us describe shortly how we parametrize the contact matrix and give some general results in this case.

The contact matrix $\Gamma$ introduces many new parameters to the model. We reduce the number of such parameters by assuming that all groups have the same activity level. That is, we assume that each individual makes on average contacts at the same rate, regardless of its group. Without loss of generality, we can assume that this average number of contacts is 1, which leads to the constraint that

23 $$\begin{aligned} p_1 \gamma _{1,1} + p_2 \gamma _{1,2} = 1,\qquad p_1 \gamma _{1,2} + p_2 \gamma _{2,2} = 1. \end{aligned}$$

In the case of a general number of groups *L* this condition would read

$$\begin{aligned} \forall \ell \in \{1, \dots , L\},\quad \sum _{\ell '=1}^L p_{\ell '} \gamma _{\ell , \ell '} = 1. \end{aligned}$$

For $L=2$, under assumption (23) and the additional constraint that contacts are symmetric, all contact matrices can be parametrized as

24 $$\begin{aligned} \Gamma = \begin{pmatrix} \frac{1}{p_1}(1-p_2\alpha ) & \alpha \\ \alpha & \frac{1}{p_2}(1-p_1\alpha ) \end{pmatrix} \end{aligned}$$

for some $\alpha \in \left[ 0,\min \left( \frac{1}{p_1},\frac{1}{p_2}\right) \right]$. The remaining degree of freedom $\alpha$, which we will refer to as the contact parameter, tunes the assortativity of the contacts:

- for $\alpha \in [0, 1)$ the population is assortative and individuals make more contacts within their own group (for $\alpha = 0$ the populations would be disconnected);
- for $\alpha = 1$ the population is well-mixed and contacts are homogeneous;
- for $\alpha \in \left( 1,\min \left( \frac{1}{p_1},\frac{1}{p_2}\right) \right]$ the population is dissortative and individuals make more contacts outside of their groups.

Under this parametrization, the endemic threshold is given by the inverse of the leading eigenvalue of the matrix

$$\begin{aligned} M=\begin{pmatrix} (1-p_2\alpha )\Sigma _{1} & p_2\alpha \Sigma _{2} \\ p_1\alpha \Sigma _{1} & (1-p_1\alpha )\Sigma _{2} \end{pmatrix}, \end{aligned}$$

where $\Sigma _1$ and $\Sigma _2$ have been defined in (21). The leading eigenvalue of this matrix corresponds to the largest root of the equation

$$\begin{aligned} \rho ^2 - ( (1-p_1\alpha )\Sigma _{2} + (1-p_2\alpha )\Sigma _{1}) \rho + (1-\alpha ) \Sigma _1\Sigma _2=0, \end{aligned}$$

given by

25 $$\begin{aligned} \rho (\alpha )&=\frac{1}{2}\left( \left( 1-p_2\alpha \right) \Sigma _{1}+\left( 1-p_1\alpha \right) \Sigma _{2}\right) \nonumber \\&\hspace{28.45274pt}+\frac{1}{2}\sqrt{\left( \left( 1-p_2\alpha \right) \Sigma _{1}+\left( 1-p_1\alpha \right) \Sigma _{2}\right) ^2-4(1-\alpha )\Sigma _{1}\Sigma _{2}}. \end{aligned}$$

Two general observations can be made at this point, which are stated in Proposition 5 below. First, when $\Sigma _1 = \Sigma _2 =:\Sigma$ the leading eigenvalue is $\rho = \Sigma$ and does not depend on the contact parameter $\alpha$. Therefore, when two groups are vaccinated in the same way ($\Sigma _1 = \Sigma _2$) and have the same activity level (equation (23) holds), the population structure does not impact the existence of an endemic equilibrium. A similar claim holds for any number of groups *L*. Second, the leading eigenvalue $\rho$ is a non-increasing function of $\alpha$ when all other parameters are fixed. This indicates that a population with less assortative contacts performs better at preventing the disease from reaching an endemic state.

#### Proposition 5

If $\Sigma _1 = \Sigma _2$, then $\rho = \Sigma _1$. Moreover, for any $\Sigma _1$ and $\Sigma _2$ the function $\alpha \mapsto \rho (\alpha )$ defined in (25) is non-increasing and convex on $\left[ 0, \min (\tfrac{1}{p_1}, \tfrac{1}{p_2}) \right]$.

#### Proof

If $\Sigma _1 = \Sigma _2$, *M* is a multiple of a stochastic matrix and the leading eigenvalue is easily seen to be $\Sigma _1$. In particular, the second part of the statement also holds in that case.

Let us now suppose that $\Sigma _1 \ne \Sigma _2$. Denoting $\Sigma = p_2 \Sigma _1 + p_1 \Sigma _2$, we write $2\rho (\alpha ) = \left( \Sigma _{1}+\Sigma _{2}-\Sigma \alpha \right) + \sqrt{z(\alpha )}$, with

$$\begin{aligned} z(\alpha )=\left( \Sigma _{1}+\Sigma _{2}-\Sigma \alpha \right) ^2+4(\alpha -1)\Sigma _{1}\Sigma _{2}. \end{aligned}$$

Thus $2\rho '(\alpha )=-\Sigma +z'(\alpha )/(2\sqrt{z(\alpha )})$ and $2\rho ''(\alpha )=\left( 2z''(\alpha )z(\alpha )-z'(\alpha )^2\right) /\left( 4z(\alpha )^{3/2}\right)$. We have

$$\begin{aligned} z'(\alpha )&=-2\Sigma \left( \Sigma _{1}+\Sigma _{2}-\Sigma \alpha \right) +4\Sigma _{1}\Sigma _{2}\\ z''(\alpha )&=2{\Sigma }^2. \end{aligned}$$

Consequently,

$$\begin{aligned} 2z''(\alpha )z(\alpha )-z'(\alpha )^2&=16\Sigma _{1}\Sigma _{2}\left( -\Sigma ^2-\Sigma _{1}\Sigma _{2} +\Sigma (\Sigma _{1}+\Sigma _{2})\right) \\&=16p_1p_2(\Sigma _{1}-\Sigma _{2})^2> 0. \end{aligned}$$

We deduce that $\alpha \mapsto \rho '(\alpha )$ is an increasing function.

By noting that

$$\begin{aligned} z(\alpha ) = \left( (1-p_2\alpha )\Sigma _{1} - (1-p_1\alpha )\Sigma _{2}\right) ^2 + 4\alpha ^2 p_1 p_2 \Sigma _1 \Sigma _2 > 0, \end{aligned}$$

we see that $\alpha \mapsto \rho (\alpha )$ is well-defined on $[0, \infty )$ and that the previous computation still holds. Moreover, $\lim _{\alpha \rightarrow \infty }\rho '(\alpha )=0$, and $\rho '$ only takes negative values. We conclude that $\rho$ is a decreasing convex function. $\square$

### Two public health applications

We now study our two situations of interest.

**Effect of vaccine hesitancy.** We model partial vaccination of the population by assuming that individuals from group 1 get vaccinated whereas individuals from group 2 do not. Let $\Sigma :=\Sigma _1$ be the mean susceptibility at the disease-free equilibrium within group 1. We think of $\Sigma$ as being fixed, corresponding to a given vaccination policy, and $p_1$ as varying depending on the fraction of the population complying with this policy. Let us assume that almost surely $\sigma (a) \rightarrow 1$ as $a \rightarrow \infty$, so that individuals immunity vanishes completely after a long-enough time. Since group 2 does not get vaccinated, the mean susceptibility in this group is set to be $\Sigma _2 = 1$.

We further assume for simplicity that the population is well-mixed ($\alpha = 1$), so that the mean offspring matrix is

$$\begin{aligned} M = \begin{pmatrix} p_1 \Sigma _1 & p_2 \\ p_1 \Sigma _1 & p_2 \end{pmatrix} \end{aligned}$$

and we can readily check that its leading eigenvalue is

$$\begin{aligned} \rho = p_1 \Sigma + p_2 = 1 - (1-\Sigma )p_1. \end{aligned}$$

Define a critical fraction $p_c$ as

26 $$\begin{aligned} p_c :=\frac{1-1/R_0}{1-\Sigma }. \end{aligned}$$

Then $p_c$ gives the critical fraction of the population that needs to be vaccinated recurrently to prevent an endemic equilibrium, that is

$$\begin{aligned} R_0\rho \le 1 \iff p_1 \ge p_c. \end{aligned}$$

There is an interesting correspondence between this formula and the well-known formula that gives the critical vaccine coverage to prevent an epidemic (Anderson and May 1982; Anderson et al. 2020). If a vaccine has an efficacy $E \in [0,1]$ (that is, if it provides a sterilizing immunity with probability *E*) then the critical fraction of the population that needs to be vaccinated to prevent an epidemic is

$$\begin{aligned} p'_c = \frac{1-1/R_0}{E}. \end{aligned}$$

In our model, the efficiency of the vaccine policy is quantified by $1-\Sigma$ which corresponds to the fraction of infections that are blocked at the stationary disease-free equilibrium if all individuals get vaccinated.

**Optimal vaccine allocation between two groups.** Consider a second situation where a fixed number of vaccine doses per unit of time is available, and these doses need to be allocated between two groups of individuals, which do not necessarily make homogeneous contacts. (The contact heterogeneity accounts for the fact that the groups may be two physically distinct locations: cities, countries, regions.) We model this situation in the following way.

Let $T_1$ and $T_2$ be the vaccination times in each group with expectations $m_1 = \mathbb {E}[T_1]$ and $m_2 = \mathbb {E}[T_2]$, and let 1/*m* be the per unit of time number of doses that can be allocated in the total population. Since the number of doses injected in group $\ell$ is $p_\ell / \mathbb {E}[T_\ell ]$, the fact that the total number of doses injected in the population is 1/*m* adds the constraint that

27 $$\begin{aligned} \frac{p_1}{\mathbb {E}[T_1]} + \frac{p_2}{\mathbb {E}[T_2]} = \frac{p_1}{m_1} + \frac{p_2}{m_2} = \frac{1}{m}. \end{aligned}$$

The set of all pairs $(m_1, m_2)$ verifying (27) for a given *m* can now be parametrized by a single parameter $\beta$ as

$$\begin{aligned} \forall \beta \in \left[ -\frac{1}{p_2},\frac{1}{p_1}\right] , \quad \frac{1}{m_1(\beta )}=\frac{1}{m}+\frac{ p_2}{m}\beta ,\quad \frac{1}{m_2(\beta )}=\frac{1}{m}-\frac{ p_1}{m}\beta . \end{aligned}$$

Under this parametrization, we can interpret $\beta$ as assessing the *fairness* of the allocation in the sense that

- when $\beta =0$, all doses are allocated evenly across the two groups;
- when $\beta >0$ population 1 is favored and if $\beta =\frac{1}{p_1}$ all the doses are allocated to population 1;
- when $\beta <0$ population 2 is favored and if $\beta =-\frac{1}{p_2}$, all the doses are allocated to population 2.

Fix some random variable *T* with $\mathbb {E}[T] = m$ and define

28 $$\begin{aligned} T_1(\beta ) = \frac{1}{1+\beta p_2} T, \qquad T_2(\beta ) = \frac{1}{1-\beta p_1} T. \end{aligned}$$

**Fig. 6:**
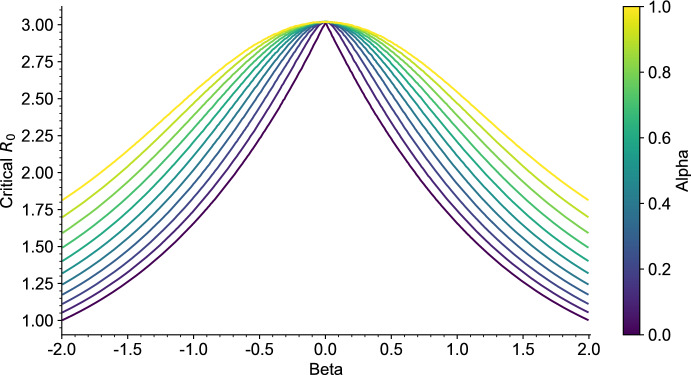
Behavior of $1/\rho$ as a function of the fairness parameter $\beta$, for different values of $\alpha$. The random variable *T* in (28) has a Gamma distribution with shape and scale parameters given by $\kappa _V$ and $\theta _V$ respectively, as in Table 1 (Appendix A). The population is assumed to be made of two groups of the same size, $p_1 = p_2 = \tfrac{1}{2}$. All other parameters are given in Table 1 (Appendix A) (color figure online)

**Fig. 7:**
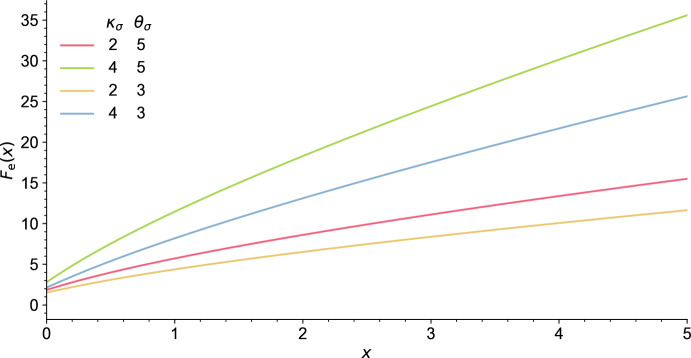
Numerical approximation of the function $F_\textrm{e}$ in (16). The susceptibility $\sigma$ is given by (40), where the parameters $\kappa _\sigma$ and $\theta _\sigma$ are given in the legend. The parameters of $T_V$ are given in Table 1 (Appendix A), and we assumed $\mathbb {E}[T_I] = 0$ (color figure online)

Then $(T_1(\beta ), T_2(\beta ))$ is a natural family of random variables verifying that $\mathbb {E}[T_\ell (\beta )] = m_\ell (\beta )$, and represents a possible allocation of the doses between the two population with fairness parameter $\beta$. We show below in Proposition 6 that, when the population is assortative ($\alpha \le 1$), the minimal eigenvalue $\rho$ is achieved at $\beta = 0$. In other words, the best possible allocation to prevent an endemic state is the fair allocation ($\beta = 0$) where individuals in both subpopulations receive the same amount of vaccines. This result is illustrated in Fig. 6.

#### Proposition 6

Let $(T_1(\beta ), T_2(\beta ))$ be as in (28) and let $\rho (\beta )$ be the largest eigenvalue of (22). Then for any fixed number of doses $m > 0$ and any $\alpha \in [0,1]$, $\beta \mapsto \rho (\beta )$ is minimal at $\beta = 0$.

#### Proof

Let us write $\rho (\alpha , \beta )$ to emphasize the dependence in the two parameters. We need to show that for any $\alpha \in [0, 1]$, $\rho (\alpha , \beta ) \ge \rho (\alpha , 0)$. From Proposition 5, for any $\alpha \in [0,1]$ and $\beta \in [\tfrac{-1}{p_2},\tfrac{1}{p_1}]$

$$\begin{aligned} \rho (\alpha , \beta ) \ge \rho (1, \beta ) :=p_1 \Sigma _1(\beta ) + p_2 \Sigma _2(\beta ), \end{aligned}$$

where for $i \in \{1, 2\}$, $\Sigma _i(\beta ) = \mathbb {E}[\phi (T_i(\beta ))] / \mathbb {E}[T_i(\beta )]$, and $\phi :a\mapsto \int _0^a\sigma (u)\textrm{d}u$. According to (27) we have

$$\begin{aligned} \frac{p_1}{\mathbb {E}[T_1(\beta )]} + \frac{p_2}{\mathbb {E}[T_2(\beta )]} = \frac{1}{\mathbb {E}[T]}, \end{aligned}$$

and we can use that $\phi$ is convex to obtain that

$$\begin{aligned} \rho (1, \beta )&= p_1 \frac{\mathbb {E}[\phi (T_1(\beta ))]}{\mathbb {E}[T_1(\beta )]} + p_2 \frac{\mathbb {E}[\phi (T_2(\beta ))]}{\mathbb {E}[T_2(\beta )]} \\&\ge \frac{1}{\mathbb {E}[T]} \mathbb {E}\Big [ \phi \Big (\frac{p_1\mathbb {E}[T]}{\mathbb {E}[T_1(\beta )]} T_1(\beta ) + \frac{p_2\mathbb {E}[T]}{\mathbb {E}[T_2(\beta )]} T_2(\beta )\Big ) \Big ] \\&= \frac{\mathbb {E}[\phi (T)]}{\mathbb {E}[T]} = \rho (1, 0) = \rho (\alpha , 0). \end{aligned}$$

In the last line, we have again used that *M* is a multiple of a stochastic matrix when $\beta = 0$, so that $\rho (\alpha , 0)$ is constant. Overall, we have shown that $\rho (\alpha , \beta ) \ge \rho (\alpha , 0)$. $\square$

## Well-posedness of the PDE system

In this section we provide proofs related to the solution of the PDE system (5). We first prove in Sect. 6.1 the existence and uniqueness of the solution. In Sect. 6.2, we prove Proposition 2, which identifies the solution as the limit of the stochastic model when the size of the population goes to infinity.

### Proof of Proposition 1

We recall that the Skorokhod space ${\mathbb {D}}(\mathbb {R}^+,\mathbb {R}^+)$ is the space of right continuous with left limits functions on $\mathbb {R}^+$ with values in $\mathbb {R}^+$ (see Billingsley 1999 for more details).

Using the definition of a weak solution, we can reformulate Eq. (5) as a set of Volterra equations. The force of infection $\Lambda$ is defined by (7),

$$\begin{aligned} \forall t \ge 0,\quad \Lambda (t) = \int _0^{\infty } \mathbb {E}[\lambda (a) \mid T_I>a]I(t,a)\textrm{d}a, \end{aligned}$$

and the mean susceptibility of the population by

29 $$\begin{aligned} \forall t \ge 0,\quad \Sigma (t)=\int _0^{\infty }\mathbb {E}_{t,a}[\sigma (a)]S(t,a)\textrm{d}a, \end{aligned}$$

where $\mathbb {E}_{t,a}[\sigma (a)]$ is given by (8). By (5), for $t\ge 0$, $I(t,0)=\Lambda (t)\Sigma (t)$ and

$$\begin{aligned} S(t,0)= \int _0^\infty \mu _I(a) I(t,a) \textrm{d}a + \int _0^\infty \mu _V(a) S(t,a) \textrm{d}a. \end{aligned}$$

An individual of age *a* at time *t* was of age 0 at time $t-a$ and no new event (recovery, vaccination, infection) occurred between $t-a$ and *t*. We thus deduce

$$\begin{aligned} \Lambda (t)&=\int _0^t\mathbb {E}[\lambda (a)] I(t-a,0)\textrm{d}a+I_0\int _t^{\infty }\mathbb {E}[\lambda (a)]h_I(a-t)\textrm{d}a \end{aligned}$$

and

$$\begin{aligned} \Sigma (t)&= \int _0^t \mathbb {E}\left[ \sigma (a) \textrm{e}^{-\int _0^a \Lambda (t-a+u) \sigma (u) \textrm{d}u} \right] e^{-\int _0^a\mu _V(u)\textrm{d}u}S(t-a,0) \textrm{d}a \\&+ \int _t^\infty \mathbb {E}\left[ \sigma (a)\textrm{e}^{-\int _{a-t}^a \Lambda (t-a+u) \sigma (u) \textrm{d}u} \right] e^{-\int _{a-t}^a \mu _V(u) \textrm{d}u} (1-I_0) h_S(a-t) \textrm{d}a \end{aligned}$$

where we used $\mathbb {E}[\lambda (a)\textbf{1}_{\{T_I>a\}}]=\mathbb {E}[\lambda (a)]$ in the expression of $\Lambda$, $\Lambda \equiv 0$ on the negative values and the vaccination rate for the initial susceptible individuals is $\mu _V(u)\textbf{1}_{\{u>a-t\}}$ for individuals of age $a\in (t,+\infty )$ in the expression of $\Sigma$ (see Sect. 2.2).

Using the same arguments for *S*(*t*, 0), we remark that the pair $(I(t,0),S(t,0); t\ge 0)$ is solution of the system of integral equations defined by, for $t\ge 0$,

30 $$\begin{aligned} \begin{aligned} x(t)&= L(t) \int _0^{\infty } \mathbb {E}\left[ \sigma (a) e^{-\int _0^a L(t-a+u)\sigma (u)\textrm{d}u} \right] e^{-\int _{(a-t)\vee 0}^a \mu _V(u) \textrm{d}u} y(t-a) \textrm{d}a, \\ y(t)&= \int _0^\infty \mu _I(a) e^{-\int _{(a-t)\vee 0}^a \mu _I(u) \textrm{d}u} x(t-a) \textrm{d}a \\&\qquad + \int _0^\infty \mu _V(a) e^{-\int _{(a-t)\vee 0}^a \mu _V(u) \textrm{d}u} \mathbb {E}\left[ e^{-\int _0^a L(t-a+u) \sigma (u) \textrm{d}u} \right] y(t-a) \textrm{d}a, \end{aligned} \end{aligned}$$

with $x(t)=I_0h_I(-t)$, $y(t)=(1-I_0)h_S(-t)$ for $t<0$ and $L(t)=\int _0^\infty \mathbb {E}[\lambda (a)]x(t-a)\textrm{d}a$ for $t\ge 0$, $L(t)=0$ for $t<0$. We observe that (*x*, *y*) is a solution of (30) if and only if

$$\begin{aligned} I(t,a)&=\textrm{e}^{-\int _{(a-t)\vee 0}^a\mu _I(u)\textrm{d}u}x(t-a)\\ S(t,a)&=\textrm{e}^{-\int _{(a-t)\vee 0}^a\mu _V(u)\textrm{d}u} \mathbb {E}\left[ \textrm{e}^{-\int _0^a L(t-a+u)\sigma (u)\textrm{d}u}\right] y(t-a) \end{aligned}$$

is a weak solution of (5).

**A priori estimates.** Let (*x*, *y*) be nonnegative functions, solution of the system (30). We introduce

$$\begin{aligned} z(t)=&\int _0^\infty \textrm{e}^{-\int _{(a-t)\vee 0}^a\mu _I(u)\textrm{d}u}x(t-a) \textrm{d}a\\&\quad +\int _0^{\infty }\mathbb {E}\left[ \textrm{e}^{-\int _0^aL(t-a+u)\sigma (u)\textrm{d}u}\right] \textrm{e}^{-\int _{(a-t)\vee 0}^a\mu _V(u)\textrm{d}u}y(t-a)\textrm{d}a, \end{aligned}$$

which can also be written, using the changes of variables $b=t-a$ on [0, *t*] and $b=a-t$ on $[0,\infty )$,

$$\begin{aligned} z(t)&=\int _0^t\textrm{e}^{-\int _0^{t-b}\mu _I(u)\textrm{d}u}x(b) \textrm{d}b+I_0\int _0^\infty \textrm{e}^{-\int _b^{b+t}\mu _I(u)\textrm{d}u}h_I(b) \textrm{d}b\\&+\int _0^{t}\mathbb {E}\left[ \textrm{e}^{-\int _0^{t-b}L(b+u)\sigma (u)\textrm{d}u}\right] \textrm{e}^{-\int _0^{t-b}\mu _V(u)\textrm{d}u}y(b)\textrm{d}b\\&+(1-I_0)\int _0^{\infty }\mathbb {E}\left[ \textrm{e}^{-\int _b^{b+t}L(u-b)\sigma (u)\textrm{d}u}\right] \textrm{e}^{-\int _b^{b+t}\mu _V(u)\textrm{d}u}h_S(b)\textrm{d}b. \end{aligned}$$

Computing the first derivative of *z*, we observe that $z'(t)=0$. Computing *z*(0), we then have, $\forall t\ge 0$,

31 $$\begin{aligned} 1= &  \int _0^\infty \textrm{e}^{-\int _{(a-t)\vee 0}^a\mu _I(u)\textrm{d}u}x(t-a) \textrm{d}a \nonumber \\ &  +\int _0^{\infty }\mathbb {E}\left[ \textrm{e}^{-\int _0^aL(t-a+u)\sigma (u)\textrm{d}u}\right] \textrm{e}^{-\int _{(a-t)\vee 0}^a\mu _V(u)\textrm{d}u}y(t-a)\textrm{d}a. \end{aligned}$$

As $\sigma \in [0, 1]$, we easily deduce from (30) and the above equation that $x(t) \le L(t)$. Moreover, by assumption $\mathbb {E}[\lambda (a)] \le \lambda _{\max }$. Consequently, by definition of *L*, we have

$$\begin{aligned} x(t)\le \lambda _{\max } \left( \int _0^t x(a)\textrm{d}a+I_0\right) . \end{aligned}$$

Using Gronwall’s Lemma, we obtain for $t \ge 0$, $x(t) \le I_0 \lambda _{\max } e^{\lambda _{\max } t}$ and thus $L(t)\le I_0 \lambda _{\max } e^{\lambda _{\max } t}$.

Let $T>0$. Since the density distribution function of $T_V$ is locally bounded, there exists $C_T > 0$ such that, $\forall t\ge 0$

$$\begin{aligned}&y(t) \le \max _{t\in [0,T]}x(t)+ C_T \int _0^t y(b)\textrm{d}b\\&\quad +\int _0^\infty \mu _I(t+b)\textrm{e}^{-\int _b^{b+t}\mu _I(u)\textrm{d}u}h_I(b)\textrm{d}b +\int _0^\infty \mu _V(t+b)\textrm{e}^{-\int _b^{b+t}\mu _V(u)\textrm{d}u}h_S(b)\textrm{d}b. \end{aligned}$$

Using Gronwall’s inequality, and assumptions of the proposition, we conclude that *y* is locally bounded on $\mathbb {R}^+$.

**Existence and uniqueness of solutions.** We now prove the uniqueness of the solution. Let $(x_1,y_1)$ and $(x_2,y_2)$ be two solutions of (30).

From (30) we have

$$\begin{aligned}&|x_1(t) - x_2(t)| \le |L_1(t) - L_2(t)| \int _0^{\infty } \mathbb {E}\left[ \sigma (a) e^{-\int _0^a L_1(t-a+u)\sigma (u)\textrm{d}u} \right] \\&\quad e^{-\int _{(a-t)\vee 0}^a \mu _V(u) \textrm{d}u} y_1(t-a) \textrm{d}a \\&\quad + L_2(t) \int _0^{\infty } \mathbb {E}\left[ \sigma (a) |e^{-\int _0^a L_1(t-a+u)\sigma (u)\textrm{d}u} - e^{-\int _0^a L_2(t-a+u)\sigma (u)\textrm{d}u} |\right] \\&\quad e^{-\int _{(a-t)\vee 0}^a \mu _V(u) \textrm{d}u} y_1(t-a) \textrm{d}a \\&\quad + L_2(t) \int _0^{\infty } \mathbb {E}\left[ \sigma (a) e^{-\int _0^a L_1(t-a+u)\sigma (u)\textrm{d}u} \right] \\&\quad e^{-\int _{(a-t)\vee 0}^a \mu _V(u) \textrm{d}u} |y_1(t-a)-y_2(t-a)| \textrm{d}a \\&\le \lambda _{\max } \int _0^t |x_1(a) - x_2(a)| \textrm{d}a\\&\quad + L_2(t) \int _0^{\infty } \int _0^a |L_1(t-a+u) - L_2(t-a+u) |\textrm{d}u\, y_1(t-a) \textrm{d}a \\&\quad + L_2(t) \int _0^t |y_1(a)-y_2(a)| \textrm{d}a. \end{aligned}$$

For the first term we have used Eq. (31) to bound the integral, and that $L_1(t) - L_2(t) = \int _0^t \mathbb {E}[\lambda (a)](x_1(a)-x_2(a)) \textrm{d}a$. For the second term we have used that $|e^{-u}-e^{-v}| \le |u-v|$. For the third time we have used that $y_1(t) = y_2(t)$ for $t < 0$. We further bound the second term by noting that

$$\begin{aligned} \int _0^\infty&\int _0^a |L_1(t-a+u) - L_2(t-a+u)| \textrm{d}u \, y_2(t-a) \textrm{d}a\\&= \int _0^t \int _{t-a}^t |L_1(v) - L_2(v)| \textrm{d}v \, y_1(t-a) \textrm{d}a\\&\quad + \int _0^t |L_1(u) - L_2(u)| \textrm{d}u \cdot \int _t^\infty y_1(t-a) \textrm{d}a \\&\le \int _0^t |L_1(u) - L_2(u)| \textrm{d}u \cdot \Big ( t \sup _{s \in [0,t]} |y_1(s)| + (1-I_0) \big ). \end{aligned}$$

Using the expression of *L*(*t*) and Fubini’s theorem we have

32 $$\begin{aligned} \int _0^t |L_1(u)-L_2(u)| \textrm{d}u \le t \lambda _{\max } \int _0^t |x_1(b)-x_2(b)| \textrm{d}b. \end{aligned}$$

Therefore, combining all the previous estimates we see that there exists $C_T$ such that for $t \le T$,

33 $$\begin{aligned} |x_1(t) - x_2(t)| \le C_T \Big (\int _0^t |x_1(s) - x_2(s)| \textrm{d}s + \int _0^t |y_1(s) - y_2(s)|\Big ) \textrm{d}s. \end{aligned}$$

In a similar way, (30) yields that

$$\begin{aligned} |y_1(t) - y_2(t)|&\le \int _0^\infty \mu _I(a) e^{-\int _{(a-t)\vee 0}^a \mu _I(u) \textrm{d}u} |x_1(t-a)-x_2(t-a)| \textrm{d}a \\&\quad + \int _0^\infty \mu _V(a) e^{-\int _{(a-t)\vee 0}^a \mu _V(u) \textrm{d}u} \\&\quad \mathbb {E}\left[ |e^{-\int _0^a L_1(t-a+u) \sigma (u) \textrm{d}u} - e^{-\int _0^a L_2(t-a+u) \sigma (u) \textrm{d}u}| \right] y_1(t-a) \textrm{d}a \\&\quad + \int _0^\infty \mu _V(a) e^{-\int _{(a-t)\vee 0}^a \mu _V(u) \textrm{d}u} \mathbb {E}\left[ e^{-\int _0^a L_1(t-a+u) \sigma (u) \textrm{d}u} \right] |y_1(t-a) - y_2(t-a)| \textrm{d}a \\&\le \int _0^t \mu _I(t-b)e^{-\int _0^{t-b} \mu _I(u) \textrm{d}u} |x_1(b)-x_2(b)| \textrm{d}b \\&\quad + \int _0^t \mu _V(a) e^{-\int _0^a \mu _V(u) \textrm{d}u} \int _{t-a}^t |L_1(b) - L_2(b)| \textrm{d}b \, y_1(t-a) \textrm{d}a \\&\quad + \int _0^t |L_1(b) - L_2(b)| \textrm{d}b \cdot (1-I_0) \int _0^\infty \mu _V(t+b) e^{-\int _b^{t+b} \mu _V(u) \textrm{d}u} h_S(b) \textrm{d}a \\&\quad + \int _0^t \mu _V(t-b) e^{-\int _{t-b}^t \mu _V(u) \textrm{d}u} |y_1(b) - y_2(b)| \textrm{d}b. \end{aligned}$$

For the first and third terms we have used that $x_1(t) = x_2(t)$ and $y_1(t) = y_2(t)$ for $t < 0$. For the second term we have split the integrals for $a > t$ and $a \le t$.

Our assumptions entail that $\mu _V(a) e^{-\int _0^a \mu _V(u) \textrm{d}u}$ and $\mu _I(a) e^{-\int _0^a \mu _I(u) \textrm{d}u}$ are bounded. Therefore, using the previous inequality, this bound, our assumption (11) on the contribution of the initial individuals together with (32) yield that there exists $C'_T$ such that, for $t \le T$,

34 $$\begin{aligned} |y_1(t) - y_2(t)| \le C'_T \int _0^t |x_1(s) - x_2(s)| + |y_1(s) - y_2(s)| \textrm{d}s. \end{aligned}$$

The estimates (33) and (34) on *x* and *y* combined with Gronwall’s inequality show that $x_1(t) = x_2(t)$ and $y_1(t) = y_2(t)$ for all $t \ge 0$, proving uniqueness of the solution to (30). The existence of a solution is proved by a classical Picard method. Let $T>0$ be fixed. For $n\ge 0$, we define by induction the sequences $(L_n)_{n\ge 0}$, $(x_n)_{n\ge 0}$ and $(y_n)_{n\ge 0}$: for $t\in [0,T]$

$$\begin{aligned} L_0(t)&=I_0\int _0^\infty \mathbb {E}[\lambda (b+t)]h_I(b)\textrm{d}b\\ x_0(t)&=I_0L_0(t)\int _0^\infty e^{-\int _{b}^{b+t}\mu _V(u)\textrm{d}u}\mathbb {E}\left[ \sigma (b+t)e^{-\int _{0}^{t}L_0(u)\sigma (u)\textrm{d}u}\right] h_I(b)\textrm{d}b\\ y_0(t)&=I_0\int _0^{\infty }\mu _I(b+t)e^{-\int _{b}^{b+t}\mu _I(u)\textrm{d}u}h_I(b)\textrm{d}b\\&\quad +(1-I_0)\mathbb {E}\left[ e^{-\int _{0}^{t}L_0(u)\textrm{d}u}\right] \int _0^\infty \mu _V(b)e^{-\int _{b}^{b+t}\mu _V(u)\textrm{d}u}h_S(b)\textrm{d}b\\ L_{n+1}(t)&=I_0\int _0^\infty \mathbb {E}[\lambda (b+t)]h_S(b)\textrm{d}b+\int _0^t\mathbb {E}[\lambda (a)]x_n(t-a)\textrm{d}a\\ x_{n+1}(t)&=I_0L_{n+1}(t)\int _0^\infty \mathbb {E}\left[ \sigma (b+t)e^{-\int _{0}^{t}L_{n+1}(u)\sigma (u)\textrm{d}u}\right] e^{-\int _{b}^{b+t}\mu _V(u)\textrm{d}u}h_I(b)\textrm{d}b\\&\quad +L_{n+1}(t)\int _0^{t}\mathbb {E}\left[ \sigma (a)e^{-\int _0^{a}L_{n+1}(t-a+u)\sigma (u)\textrm{d}u}\right] e^{\int _{0}^{a}\mu _V(u)\textrm{d}u}y_n(t-a)\textrm{d}a\\ y_{n+1}(t)&=I_0\int _0^{\infty }\mu _I(b+t)e^{-\int _{b}^{b+t}\mu _I(u)\textrm{d}u}h_I(b)\textrm{d}b\\&\quad +(1-I_0)\mathbb {E}\left[ e^{-\int _{0}^{t}L_{n+1}(u)\textrm{d}u}\right] \int _0^\infty \mu _V(b+t)e^{-\int _{b}^{b+t}\mu _V(u)\textrm{d}u}h_S(b)\textrm{d}b\\&\quad +\int _0^{t}\mu _I(a)e^{-\int _{0}^{a}\mu _I(u)\textrm{d}u}x_n(t-a)\textrm{d}a\\&\quad +\int _0^t\mu _V(a)e^{-\int _{0}^a\mu _V(u)\textrm{d}u}\mathbb {E}\left[ e^{-\int _{0}^aL_{n+1}(t-a+u)\textrm{d}u}\right] y_n(t-a)\textrm{d}a. \end{aligned}$$

By iteration and using Eq. (34), we prove that

$$\begin{aligned} &  |x_{n+1}(t)-x_n(t)|+|y_{n+1}(t)-y_n(t)| \\ &  \quad \le C_T^n\int _0^t\int _0^{t_{n-1}}\cdots \int _0^{t_1}|x_{1}(a)-x_0(a)|+|y_{1}(a)-y_0(a)|\textrm{d}a\textrm{d}t_1\ldots \textrm{d}t_{n-1} \end{aligned}$$

and then, denoting by $\Vert .\Vert _{[0,T]}$ the uniform distance on the interval [0, *T*],

$$\begin{aligned} \Vert x_{n+1}-x_n\Vert _{[0,T]}+\Vert y_{n+1}-y_n\Vert _{[0,T]}\le \frac{C_T^nT^n}{n!}\left( \Vert x_{1}-x_0\Vert _{[0,T]}+\Vert y_{1}-y_0\Vert _{[0,T]}\right) . \end{aligned}$$

The upper-bound is the general term of a converging series, and we deduce that the sequences $(x_n)_{n\ge 0}$ and $(y_n)_{n\ge 0}$ converge on the interval [0, *T*] to a solution of (30). We proved existence and uniqueness of a solution to (30) on the interval [0, *T*] for any $T>0$, we then deduce the existence and uniqueness on $\mathbb {R}^+$.

### Proof of Proposition 2

We recall that $(\lambda ^*(t), \sigma ^*(t),A^*(t), C^*(t))$ is the solution to the McKean–Vlasov Eq. (4). We start by deriving the equation for $I(t, \cdot )$. Let us compute, for some test function $\varphi$,

$$\begin{aligned} \mathbb {E}\big [ \varphi (A^*(t)) \textbf{1}_{\{C^*(t) = I\}} \big ] = \sum _{k \ge 0} \mathbb {E}\big [ \textbf{1}_{\{K^*(t) = k, C^*_{k} = I\}} \varphi (t-\tau ^*_{k}) \big ]. \end{aligned}$$

We have that

$$\begin{aligned} \{ K^*(t) = k \} \cap \{ C^*_{k} = I \} = \{ \tau ^*_{k} \le t < \tau ^*_{k}+T_{I,k} \} \cap \{ C^*_{k} = I \}. \end{aligned}$$

For $k = 0$, by our choice of initial condition, see [IC] in Sect. 2.1,

$$\begin{aligned} \mathbb {E}\big [ \varphi (A^*(t)) \textbf{1}_{\{C^*(t) = I, K^*(t)=0\}} \big ] = I_0 \int _0^\infty h_I(a) \varphi (t+a) \exp \Big ( -\int _a^{t+a} \mu _I(u) \textrm{d}u \Big ) \textrm{d}a. \end{aligned}$$

For $k \ge 1$, $T_{I,k}$ is independent of $\tau ^*_{k}$ and $C^*_{k}$ so that

$$\begin{aligned} \mathbb {E}\big [ \varphi (A^*(t))&\textbf{1}_{\{C^*(t) = I, K^*(t) \ge 1\}} \big ] \\&= \sum _{k \ge 1} \mathbb {E}\Big [ \textbf{1}_{\{\tau ^*_{k} \le t, C^*_k = I\}} \varphi (t-\tau ^*_{k}) \exp \Big (- \int _0^{t-\tau ^*_{k}} \mu _I(u) \textrm{d}u \Big ) \Big ]\\&= \mathbb {E}\Big [ \int _{[0,t]} \varphi (t-a) \exp \Big (- \int _0^{t-a} \mu _I(u) \textrm{d}u \Big ) P_I(\textrm{d}a) \Big ] \end{aligned}$$

where $P_I$ is the point process of infection times, which is the random measure on $[0, \infty )$ defined as

$$\begin{aligned} P_I(B) = \sum _{k \ge 1} \textbf{1}_{\{\tau ^*_{k} \in B\}} \textbf{1}_{\{C^*_{k} = I\}}. \end{aligned}$$

Since infections occur at rate $\Lambda ^*(t) \sigma ^*(t)$ for $t \ge 0$, the density of the intensity measure of $P_I$ is $\Lambda ^*(t) \mathbb {E}[\sigma ^*(t)]$ so that

$$\begin{aligned} &  \mathbb {E}\big [ \varphi (A^*(t)) \textbf{1}_{\{C^*(t) = I\}} \big ] = \int _0^t \Lambda ^*(a) \Sigma ^*(a) \varphi (t-a) \exp \Big (- \int _0^{t-a} \mu _I(u) \textrm{d}u \Big ) \textrm{d}a \\ &  \quad + I_0 \int _0^\infty h_I(a) \varphi (t+a) \exp \Big (- \int _a^{t+a} \mu _I(u) \textrm{d}u \Big ) \textrm{d}a \end{aligned}$$

with $\Sigma ^*(t) = \mathbb {E}[\sigma ^*(t)]$. This shows that the density of $A^*(t)$ on $\{C^*(t) = I\}$ is

$$\begin{aligned} \forall a \le t,\quad I(t,a) = \Lambda ^*(t-a) \Sigma ^*(t-a) \exp \Big (- \int _0^a \mu _I(u) \textrm{d}u \Big ) \end{aligned}$$

and

$$\begin{aligned} \forall a \ge t,\quad I(t,a) = I_0 h_I(a-t) \exp \Big (- \int _{a-t}^a \mu _I(u) \textrm{d}u \Big ) \end{aligned}$$

which is the weak solution to

$$\begin{aligned} \partial _t I(t,a) + \partial _a I (t,a)&= -\mu _I(a) I(t,a)\\ I(t,0)&= \Lambda ^*(t) \Sigma ^*(t) \\ I(0,a)&= I_0 h_I(a). \end{aligned}$$

We obtain the first part of the PDE limit (5) ($\Sigma ^*$ will be identified at the end of the proof). We now turn to the density of susceptible individuals. As previously

$$\begin{aligned} \{ K^*(t) = k \} \cap \{ C^*_{k} = S \} = \{ \tau ^*_{k} \le t < \tau ^*_{k}+T_{V,k} \wedge Z^*_{k} \} \cap \{ C^*_{k} = S \}, \end{aligned}$$

so that for $k = 0$, recalling our initial condition [IC] and the expression for the reinfection time $Z^*_{k}$ given by (2) with a $\Lambda ^*$ instead of $\Lambda ^N$ yields that

$$\begin{aligned} &  \mathbb {E}\big [ \varphi (A^*(t)) \textbf{1}_{\{C^*(t) = S, K^*(t) = 0\}} \big ] \\ &  \quad = (1-I_0) \int _0^\infty h_S(a) \varphi (t+a) e^{- \int _a^{t+a} \mu _V(u) \textrm{d}u} \mathbb {E}\Big [e^{-\int _a^{t+a} \Lambda ^*(u-a) \sigma (u) \textrm{d}u}\Big ] \textrm{d}a. \end{aligned}$$

Indeed, from (2) and by independence, we notice that

$$\begin{aligned}&\mathbb {P}(\min (T_{V,0}, Z^*_{0})>t+a \mid T_{V,0}>a)\\&\quad =\mathbb {P}(T_{V,0}>t+a \mid T_{V,0}>a)\mathbb {P}( Z^*_{0}>t+a)\\&\quad =e^{- \int _a^{t+a} \mu _V(u) \textrm{d}u}\,\mathbb {P}\left( \int _0^{t+a}\Lambda ^*(-a+u)\sigma _0(u)\textrm{d}u<E_{0}\right) \\&\quad =e^{- \int _a^{t+a} \mu _V(u) \textrm{d}u} \mathbb {E}\Big [e^{-\int _a^{t+a} \Lambda ^*(u-a) \sigma (u) \textrm{d}u}\Big ]. \end{aligned}$$

Similarly, for $k \ge 1$, using the independence of the various variables

$$\begin{aligned} &  \mathbb {E}\big [ \varphi (A^*(t)) \textbf{1}_{\{C^*(t) = S, K^*(t) \ge 1\}} \big ] \\ &  \quad = \mathbb {E}\Big [ \int _{[0,t]} \varphi (t-a) e^{- \int _0^{t-a} \mu _V(u) \textrm{d}u} \mathbb {E}\Big [e^{-\int _0^{t-a} \Lambda ^*(a+u)\sigma (u) \textrm{d}u}\Big ] P_S(\textrm{d}a) \Big ] \end{aligned}$$

with the point process $P_S$ defined on $(0, \infty )$ as

$$\begin{aligned} P_S(B) = \sum _{k \ge 1} \textbf{1}_{\{\tau ^*_{k} \in B\}} \textbf{1}_{\{C^*_{k} = S\}}. \end{aligned}$$

The intensity of $P_S$ has a density that we denote by $p_S$. Recall Eq. (8), the next step is to note that

35 $$\begin{aligned} \mathbb {E}\Big [ e^{-\int _0^a \Lambda ^*(t-a+u) \sigma (u) \textrm{d}u} \Big ] = e^{-\int _0^a \Lambda ^*(t-a+u) \mathbb {E}_{t-a+u,u}[\sigma (u)] \textrm{d}u}. \end{aligned}$$

This is equivalent to showing that

$$\begin{aligned} \int _0^a \Lambda ^*(t-a+u) \mathbb {E}_{t-a+u,u}[\sigma (u)] \textrm{d}u = - \log \mathbb {E}\Big [ e^{-\int _0^a \Lambda ^*(t-a+u) \sigma (u) \textrm{d}u} \Big ]. \end{aligned}$$

Applying the operator $\partial _t + \partial _a$ to both sides leads to

$$\begin{aligned} \Lambda ^*(t) \mathbb {E}_{t,a}[\sigma (a)] = \Lambda ^*(t) \mathbb {E}\Big [ \sigma (a) e^{-\int _0^a \Lambda (t-a+u) \sigma (u) \textrm{d}u} \Big ] \, \Big /\,\mathbb {E}\Big [ e^{-\int _0^a \Lambda (t-a+u) \sigma (u) \textrm{d}u} \Big ] \end{aligned}$$

and we recover the expression for $\mathbb {E}_{t,a}$, see (8).

Therefore, combining the previous expressions,

$$\begin{aligned}&\mathbb {E}\big [ \varphi (A^*(t)) \textbf{1}_{\{C^*(t) = S\}} \big ]= (1-I_0) \int _t^\infty h_S(a-t) \varphi (a) e^{- \int _{a-t}^a \mu _V(u) \textrm{d}u} \mathbb {E}\Big [e^{-\int _{a-t}^a \Lambda ^*(t-a+u) \sigma (u) \textrm{d}u}\Big ] \textrm{d}a \\&\quad + \int _0^t \varphi (a) e^{- \int _0^a \mu _V(u) \textrm{d}u-\int _0^a \Lambda ^*(t-a+u) \mathbb {E}_{t-a+u,u}[\sigma (u)] \textrm{d}u} p_S(t-a) \textrm{d}a \end{aligned}$$

and we recover the weak solution of

$$\begin{aligned} \partial _t S(t,a) + \partial _a S(t,a)&= -\mu _V(a)S(t,a) - \Lambda ^*(t) \mathbb {E}_{t,a}[\sigma (a)] S(t,a)\\ S(t,0)&= p_S(t)\\ S(0, a)&= (1-I_0) h_S(a). \end{aligned}$$

Our last task is to compute $\Lambda ^*(t)$, $\Sigma ^*(t)$, and $p_S(t)$. For the latter quantity, by construction of the process, for any $t \ge 0$,

$$\begin{aligned} &  \mathbb {E}\big [ P_S([t, t+\textrm{d}t]) \mid A^*(t), C^*(t) \big ] \\ &  \quad = \left( \mu _I(A^*(t)) \textbf{1}_{\{C^*(t) = I\}} + \mu _V(A^*(t)) \textbf{1}_{\{C^*(t) = S\}}\right) \textrm{d}t. \end{aligned}$$

Therefore

$$\begin{aligned} \forall t \ge 0, \quad p_S(t) = \int _0^\infty \mu _I(a) I(t,a) \textrm{d}a + \int _0^\infty \mu _V(a) S(t,a) \textrm{d}a. \end{aligned}$$

Similarly, by conditioning on $A^*(t)$,

$$\begin{aligned} \forall t \ge 0,\quad \Lambda ^*(t) = \mathbb {E}[\lambda ^*(t)] = \int _0^\infty I(t,a) \mathbb {E}\big [ \lambda (a) \mid T_I > a\big ] \textrm{d}a \end{aligned}$$

and

$$\begin{aligned} \forall t \ge 0,\quad \Sigma ^*(t)&= \mathbb {E}[\sigma ^*(t)] = \int _0^\infty S(t,a) \mathbb {E}[\sigma (a) \mid T_V> a, Z > a] \textrm{d}a \\&= \int _0^\infty S(t,a) \mathbb {E}_{t,a}[\sigma (a)]\textrm{d}a. \end{aligned}$$

## Summary and discussion

**Summary.** In this work we have proposed an individual-based model to study the effect of recurrent vaccination on the establishment of an endemic equilibrium, in a population with waning immunity. Our model incorporates memory effects both for the transmission rate during an infection and for the subsequent immunity, and takes into account the stochasticity at the individual level for these two processes. By deriving the large population size limit of the model and analysing its equilibria, we have obtained a simple criterion for the existence of an endemic equilibrium. This criterion depends jointly on the shape of the rate of immunity loss and on the distribution of the time between two booster doses. In other words, in the context of recurrent vaccination and waning immunity, what drives the result of a vaccination-policy is a combination of the efficiency of the vaccine itself at blocking transmissions, and of the way in which booster doses are distributed in the population. The expression we obtain relates directly to the average immunity level maintained by vaccinating recurrently the population, which is a relation that we expect to hold for a broad class of models with similar characteristics.

One general public health conclusion that we can draw from our work is that, for the same average number of vaccine doses available, vaccination strategies where the time between booster shots are more evenly spaced (at the individual level) perform better at blocking transmissions. A similar conclusion was reached recently by Khalifi and Britton (2024) for a related model. Intuitively, irregularly spread booster doses lead to some longer time intervals without vaccination, and the resulting high susceptibility allows the disease to spread more efficiently. Deriving further conclusions from our model would require to add some restrictions on the distributions of $T_V$ and $\sigma$ that would reflect the characteristics of a particular disease and vaccine.

Finally, we have studied two specific situations in more details. First, we have computed an expression for the critical fraction of the population required to adhere to the vaccination policy to eradicate the disease (see (26)). This expression is reminiscent of a well-known threshold for preventing an endemic state with an imperfect vaccine (Anderson and May 1985). In the context of recurrent vaccination, the efficiency of the vaccine is replaced by the average susceptibility obtained by vaccinating individuals in the absence of disease. Second we have studied the consequences of uneven vaccine access in a population, and concluded that fair vaccine allocation is the optimal strategy to prevent endemicity (see Proposition 6).

**Model assumptions.** Our model is formulated in terms of infectiousness and susceptibility, which are two phenomenological quantities that result from the complex interaction between the pathogen and the host immune system. If this interaction were modeled explicitly as in many existing works on viral dynamics (Heffernan and Keeling 2008, 2009; Ashish Goyal et al. 2020; Néant et al. 2021), infectiousness would relate to the viral load, and susceptibility to the level of immune cells or circulating antibodies. Since we have left the susceptibility and infectiousness be general random functions, our model should encompass many possible such host-pathogen models. There are two assumptions that we have made about $\lambda$ and $\sigma$ that could be easily relaxed mathematically, but would lead to a more complicated model. First, we assumed that the susceptibility curve following an infection is independent of the infectiousness curve during that infection. A typical situation where this assumption would fail is if a more severe infection leads both to a larger infectiousness and to a higher level of immunity (and thus a lower susceptibility). Second, we assumed that infection and vaccination lead to the same susceptibility in distribution. We expect a law of large number similar to Theorem 1 to hold if these assumptions are relaxed, with a similar criterion for the existence of an endemic equilibrium and mild modifications of the limit equations. However, our mathematical results rely crucially on the strong assumption that individuals (and thus their immune system) keep no memory of past infections or vaccinations: at each reinfection or vaccination, the subsequent infectiousness and susceptibility are sampled independently and according to the same law. In particular, the expression (18) for the herd immunity threshold follows from the fact that vaccinations form a renewal process, which is a consequence of this absence of memory from past vaccinations. Relaxing this assumption would require a completely different approach to our problem. Nonetheless, the key quantity in our model is the stationary susceptibility of a typical individual, obtained by letting an individual get vaccinated only for a long period of time. It might be the case that other models displaying a similar stationary behavior have the same qualitative properties as the one investigated here.

The persistence of a disease requires a continual replenishment of susceptible individuals to sustain the epidemic. In our model, this influx of susceptibility comes exclusively from waning immunity. Two other important causes for an increase in susceptibility that we have neglected are the birth of new individuals with no immunity and the pathogen evolution to escape immunity. We expect that, as long as the population size is stable and newborns start being vaccinated rapidly, demographic effects (that we have neglected by considering a closed population) should not impact our conclusions to a large extent. The key quantity that controls the establishment of an endemic equilibrium in our model is the level of population immunity in the absence of disease, which should be mostly driven by vaccination if the typical time between two vaccine doses is small compared to the lifetime of individuals. Taking into account pathogen evolution is, however, a more challenging task that would require further investigation and modeling. Though, note that a model structured by time-since-recovery similar to the one we consider here has been proposed to study the increase in susceptibility due to antigenic drift in influenza strains (Pease 1987).

**Discussion.** In the second half of our work, we have used the endemic threshold $1/\Sigma$ to quantify the efficiency of a given vaccination policy. This criterion has the advantage of having a clear interpretation (in terms of the average level of susceptibility maintained by vaccination), of being easy to compute and of depending only on a few average quantities of the model: the basic reproduction number $R_0$, the expected susceptibility at a given time $\mathbb {E}[\sigma (a)]$, and the distribution of $T_V$. Another interesting indicator of the impact of a vaccination policy is the so-called endemic level, defined as the prevalence of the disease at the endemic equilibrium. Ultimately, it is this endemic level that public health measures try to control, to reduce the burden of the disease in the population. In our model, when an endemic equilibrium exists, the endemic level is given by $x \mathbb {E}[T_I] / R_0$, where *x* solves $F_\textrm{e}(x) = R_0$ as in Proposition 4. The endemic level is therefore only implicitly defined, which makes it more complicated to study both analytically and numerically. Investigating the impact of the vaccination policy on the endemic level, though important, would therefore require further work, and the conclusions reached in Sect. 5.4 could be altered by using this endemic level as a criterion for the efficiency of vaccination instead of the endemic threshold. Note that the question of the impact of the way immunity is waning on the endemic level has been the subject of a recent study (El Khalifi and Britton 2023).

The simplest epidemic models consider the spread of a disease in a population made of identical individuals, that are mixing homogeneously: individuals are equally susceptible to the disease, equally infectious once infected, and contacts are equally likely to occur between any pair of individuals in the population (Britton et al. 2019, Part I). Many works have studied the epidemiological consequences of relaxing these assumptions, to account for some of the heterogeneity which is observed in human populations (Britton et al. 2020; Brauer 2008; Magal et al. 2016; Andreasen 2011; David 2018). In a similar way, we have added some heterogeneity to our model in Sect. 5 by assuming that the population is subdivided into a finite number of groups, that contacts between groups are heterogeneous and that individuals in different groups get vaccinated according to different distributions of $T_V$. Since our focus is the impact of inhomogeneous vaccination on endemicity, we have assumed that all groups have the same activity level ($\Gamma \times {{\,\textrm{Diag}\,}}(p)$ is a multiple of a stochastic matrix), and that they sample their infectiousness and susceptibility curves from the same distribution. Our model could be easily extended to allow the distribution of the infectiousness and susceptibility curves to depend on the group, and to general contact matrices $\Gamma$. Using the same heuristic arguments as in Sect. 5.2, we can derive a criterion for the existence of an endemic equilibrium in terms of the leading eigenvalue of a next-generation matrix similar to (22). However, although it is possible to derive such an expression, the joint effect of heterogeneous infectiousness, susceptibility, contact rates and vaccination rates on this criterion is extremely complex, but would be a very interesting avenue for future work.

Finally, following the tradition of classical epidemiology models, we have considered the groups as being fixed during the course of the epidemic. Although this assumption might be realistic if groups model “physical” heterogeneities (age classes on a short time-scale, spatial locations), it becomes simplistic when groups model human behavior (compliance to public health measures, vaccine hesitancy). In the latter situation, the group to which an individual belongs can possibly change and is influenced by many factors, including perceived risks of vaccine adverse events, disease prevalence, or available information. Modeling such effects appropriately is a challenging task that is the focus of behavioral epidemiology (Bauch 2005; d’Onofrio et al. 2011; Antonella Lupica et al. 2020; Manfredi and D’Onofrio 2013). Incorporating such effects in the current model is an interesting avenue for future work.

It is interesting to compare our expression for the endemic threshold to that recently obtained in Forien et al. (2022), for a similar model but without vaccination. In Forien et al. (2022) it is shown that, in the absence of vaccination and using our notation, an endemic equilibrium exists if and only if

36 $$\begin{aligned} \frac{1}{R_0} \mathbb {E}\Big [ \frac{1}{\sigma _*} \Big ] < 1, \end{aligned}$$

where $\sigma _* :=\lim _{a \rightarrow \infty } \sigma (a)$. The corresponding expression with vaccination that we have obtained is

37 $$\begin{aligned} \frac{\mathbb {E}[T_V]}{R_0 \mathbb {E}\Big [ \int _0^{T_V} \sigma (a) \textrm{d}a \Big ]} < 1. \end{aligned}$$

By letting $T_V \rightarrow \infty$, we expect that our model converges to the model considered in Forien et al. (2022) where no vaccination is taken into account. However, in the limit $T_V \rightarrow \infty$ our expression for the endemicity criterion becomes

38 $$\begin{aligned} \frac{1}{R_0 \mathbb {E}[\sigma _*]} < 1. \end{aligned}$$

Note the surprising discrepancy between (36) and (38). This apparent contradiction can be resolved by noting that both expressions are specific cases of a more general formula. Let $\zeta (u)$ denote the susceptibility of a typical individual at age-of-infection *u*, that is, *u* unit of time after its last infection, regardless of how many times it has been vaccinated since then. Then, mimicking the computations of Sect. 3.1 would suggest that the correct threshold for the existence of an endemic equilibrium is given by

39 $$\begin{aligned} \frac{1}{R_0} \mathbb {E}\Big [ \lim _{a \rightarrow \infty } \frac{1}{\frac{1}{a}\int _0^a \zeta (u) \textrm{d}u} \Big ] < 1, \end{aligned}$$

provided that the limit in the expectation exists. In the absence of vaccination, $\zeta (u) = \sigma (u)$ and we recover (36). In the presence of vaccination, letting $\sigma _i$ and $T_i$ be i.i.d., we have

$$\begin{aligned} \forall u \ge 0,\quad \zeta (u) = \sigma _i(u - (T_1+\dots +T_i)) \textbf{1}_{\{T_1 + \dots + T_i \le u < T_1 + \dots + T_{i+1}\}} \end{aligned}$$

and classical results on renewal processes show that we recover (38). We believe that (39) should give the correct threshold for the existence of an endemic equilibrium for a broader class of models.

## Data Availability

Data sharing not applicable to this article as no datasets were generated or analysed during the current study.

## Numerical simulations

### Model parametrization

In all simulations, we assumed that the laws ${\mathcal {L}}_\lambda$, ${\mathcal {L}}_\sigma$ and ${\mathcal {L}}_V$ have the following form.

The law ${\mathcal {L}}_\lambda$ depends on four parameters: two shape parameters $\kappa _I, \kappa '_I$, one scale parameter $\theta _I$ and one parameter $R_0$ for the total mass. Then, we define two independent random variables

$$\begin{aligned} &  T_I \sim \textrm{Gamma}(\kappa _I + \kappa '_I, \theta _I) :=\frac{x^{\kappa _I+\kappa '_I-1}\,e^{-x/\theta _I}}{\Gamma (\kappa _I+\kappa '_I) \theta _I^{\kappa _I+\kappa '_I}} \textrm{d}x, \\ &  M \sim \textrm{Exponential}(R_0) :=\frac{e^{-x/R_0}}{R_0} \textrm{d}x \end{aligned}$$

and we set

$$\begin{aligned} \forall a < T_I,\quad \lambda (a) = \frac{M}{T_I \textrm{B}(\kappa _I,\kappa '_I)} \big (\tfrac{a}{T_I}\big )^{\kappa _I-1} \big (1-\tfrac{a}{T_I}\big )^{\kappa '_I-1} \end{aligned}$$

where $\textrm{B}(\kappa , \kappa ')$ is the Beta function. In words, $\lambda$ has the shape of the density of a Beta distribution, stretched by a random factor $T_I$, and with random total mass *M*. Note that we do have

$$\begin{aligned} \mathbb {E}\Big [ \int _0^{T_I} \lambda (a) \textrm{d}a \Big ] = R_0. \end{aligned}$$

This choice of parametrization was chosen so that $\mathbb {E}[\lambda (a)]$ is easy to compute by standard properties of Beta and Gamma distributions.

**Table 1:** Default parameter values for the simulations

Parameter	Description	Value
$\kappa _I, \kappa _I'$	Shape parameters of $\lambda$	3
$\theta _I$	Scale parameter of $\lambda$	2
$\kappa _V$	Shape parameter of $T_V$	4
$\theta _V$	Scale parameter of $T_V$	3
$\kappa _\sigma$	Shape parameter of $\sigma$	5
$\theta _\sigma$	Scale parameter of $\sigma$	2
$h A^*$	Maximal age	150

In all simulations, except those of Fig. 7, the susceptibility curve $\sigma$ is deterministic and depends on two parameters: a shape parameter $\kappa _\sigma$ and a scale parameter $\theta _\sigma$. Let $G(x; \kappa , \theta )$ be the cumulative distribution function of a $\textrm{Gamma}(\kappa , \theta )$ random variable, evaluated at *x*, that is,

$$\begin{aligned} \forall x \ge 0,\quad G(x; \kappa , \theta ) = \mathbb {P}(X \le x), \qquad X \sim \textrm{Gamma}(\kappa , \theta ). \end{aligned}$$

We assume that

$$\begin{aligned} \forall a \ge 0,\quad \sigma (a) = G(a; \kappa _\sigma , \theta _\sigma ). \end{aligned}$$

In the simulations of Fig. 7 only, we assume that $\sigma$ is stochastic. More precisely, we obtain a random $\sigma$ by letting the parameters of the Gamma distribution be themselves random (and Gamma distributed with mean $\kappa _\sigma$ and $\theta _\sigma$), that is,

40 $$\begin{aligned} &  \forall a \ge 0,\quad \sigma (a) = G(a; K_\sigma , \Theta _\sigma ),\nonumber \\ &  K_\sigma \sim \textrm{Gamma}(2, \tfrac{\kappa _\sigma }{2}), \qquad \Theta _\sigma \sim \textrm{Gamma}(2, \tfrac{\theta _\sigma }{2}). \end{aligned}$$

The law of the vaccination duration $T_V$ also depends on one shape parameter $\kappa _V$ and one scale parameter $\theta _V$. We simply assume that $T_V$ follows a Gamma distribution with these parameters:

$$\begin{aligned} T_V \sim \textrm{Gamma}(\kappa _V, \theta _V). \end{aligned}$$

For the heterogeneous model, we use a similar parametrization. We suppose that for each $i \in \{1, \dots , L\}$ we have

$$\begin{aligned} T_i \sim \textrm{Gamma}(\kappa _i, \theta _i) \end{aligned}$$

where $\kappa _i, \theta _i$ are the shape and scale parameters of $T_i$.

### Initial condition

We selected an initial conditions close to the endemic equilibrium to speed up the convergence to it, and make the transient behavior of the system shorter. Precisely, for all simulations we used

$$\begin{aligned} &  \forall a \ge 0,\qquad h_I(a) = \exp \Big ( - \int _0^a \mu _I(u) \textrm{d}u \Big ) / \mathbb {E}[T_I],\\ &  h_S(a) = \exp \Big ( - \int _0^a \mu _V(u) \textrm{d}u \Big ) / \mathbb {E}[T_V]. \end{aligned}$$

For the heterogeneous model, we set the initial age structure in each group as above, using the vaccination time distribution $T_i$ of the corresponding group for $h_S$.

### Numerical approximations of the PDE

We approximate the solution $(S(t,a), I(t,a);\, t,a \ge 0)$ of the PDE (5) on a finite lattice ${\mathcal {G}} = \{ (\eta i, \eta k);\, i \in \{0, \dots , T^*\}, k \in \{0,\dots , A^*\} \}$ with a small discretization step $\eta > 0$, using the method of characteristics.

The approximation is a vector $(I_{i,k}, S_{i,k};\, (i,k) \in {\mathcal {G}})$ defined inductively as follows. We let the time boundary condition be

$$\begin{aligned} \forall k \in \{0,\dots , K^*\},\qquad I_{0,k} = I_0 \frac{h_I(\eta k)}{\eta \sum _{k=0}^{K^*} h_I(\eta k)}, \qquad S_{0,k} = (1-I_0) \frac{h_S(\eta k)}{\eta \sum _{k=0}^{K^*} h_S(\eta k)}, \end{aligned}$$

Moreover, we define the approximation of the force of infection as

$$\begin{aligned} \forall i \le T^*,\quad \Lambda _i = \eta \sum _{k=0}^{A^*} \mathbb {E}[\lambda (\eta k)] I_{i,k}. \end{aligned}$$

**Table 2:** Additional parameter values for the simulations of the heterogeneous model

Parameter	Description	Value
$\kappa _1$, $\kappa _2$, $\kappa _3$	Shape parameters of $T_1$, $T_2$, $T_3$	10, 5, 6
$\theta _1$, $\theta _2$, $\theta _3$	Scale parameters of $T_1$, $T_2$, $T_3$	2, 4, 1.5
$p_1$, $p_2$, $p_3$	Proportions of individuals in each groups	0.1, 0.3, 0.6
$\Gamma$	Contact rates matrix	$$\begin{pmatrix} 1.5 & 1 & 0.2 \\ 1 & 2 & 1.5 \\ 0.2 & 0.5 & 3 \end{pmatrix}$$

For the age boundary condition we let for $i \in \{0, \dots , T^*-1\}$

$$\begin{aligned} I_{i+1, 0}= &  \Lambda _i \cdot \Big ( \eta \sum _{k=0}^{A^*} \sigma (\eta k) S_{i,k} \Big ),\\ S_{i+1, 0}= &  \eta \sum _{k=0}^{A^*} \big ( \mu _I(\eta k) I_{i,k} + \mu _V(\eta k) S_{i,k} \big ). \end{aligned}$$

Finally, for $i \in \{0, \dots T^*-1\}$ and $k \in \{0, \dots , K^*-1\}$ we define

$$\begin{aligned} I_{i+1,k+1}= &  I_{i, k} \big ( 1 - \eta \mu _I(\eta k) \big ) + \textbf{1}_{\{k+1 = K^*\}} I_{i, K^*} \big ( 1 - \eta \mu _I(\eta K^*) \big ) \\ S_{i+1,k+1}= &  S_{i, k} \big ( 1 - \eta ( \mu _V(\eta k) + \Lambda _i \sigma (\eta k) ) \big )\\ &  + \textbf{1}_{\{k+1 = K^*\}} S_{i, K^*} \big ( 1 - \eta ( \mu _V(\eta K^*) + \Lambda _i \sigma (\eta K^*) ) \big ). \end{aligned}$$

Note that the second term in each expression corresponds to a reflecting age boundary at $k = K^*$.

The solution of the PDE (20) for heterogeneous contacts is approximated using a straightforward adaptation of the above scheme.
